# A dexterous soft hand exoskeleton restores intentional grasping in individuals with severe hand impairment

**DOI:** 10.1038/s42256-026-01263-3

**Published:** 2026-06-23

**Authors:** John Nassour, Nicolas Berberich, Daniel Utpadel-Fischler, Tobias Wächter, Gordon Cheng

**Affiliations:** 1 Institute for Cognitive Systems, Technical University of Munich, Munich, Germany; 2 Department of Neurology, Klinikum Rechts der Isar, School of Medicine, Technical University of Munich, Munich, Germany; 3 Department of Neurology, Centre of Movement Disorders, Centre of Rehabilitation Passauer Wolf, Bad Goegging, Germany; 4 Department of Neurodegeneration, University of Tübingen, Tübingen, Germany; 5 Hertie-Institute for Clinical Brain Research, Tübingen, Germany; 6 Center of Competence NeuroEngineering, Technical University of Munich, Munich, Germany

**Keywords:** Quality of life, Biomedical engineering

## Abstract

Soft hand exoskeletons have emerged as promising assistive devices for individuals with impaired hand function. However, most existing systems provide limited dexterity and primarily target users with moderate hand ability, leaving individuals with severe hand paralysis without effective solutions for reliable grasping of diverse objects. Here we report the translational development of a lightweight, textile-based soft robotic exoskeleton glove with wrist dorsiflexion and an active opposable and abductable thumb, designed to restore hand function in a patient with severe right-hand impairment due to amyotrophic lateral sclerosis. We followed a co-creation approach, enhancing dexterity by increasing hand articulations based on patient needs. Furthermore, to enhance the patient’s sense of control, a non-invasive surface electromyography-based grasp predictor (97% sensitivity) was combined with motion data and machine learning-based error correction to compensate for weak, noisy muscle signals, compared with healthy controls (*n* = 15). The exoskeleton enabled the patient to grasp objects, achieve a Box-and-Blocks Test score of 5 and perform meaningful tasks, including feeding himself. We further validated the exoskeleton in patients with stroke (*n* = 6). While exoskeleton assistance on average reduced Action Research Arm Test scores of moderately impaired patients by 9, severely impaired patients scored 17 points higher when using the exoskeleton. These results indicate that the dexterous soft hand exoskeleton is particularly effective for individuals with severe to near-complete hand paralysis, while its utility for patients with moderate residual function is limited and task dependent.

## Abstract (web summary)

Nassour, Berberich and colleagues present a soft robotic hand exoskeleton that restores grasping ability in individuals with severe hand paralysis, enabling meaningful tasks such as feeding. A lightweight textile glove with wrist dorsiflexion and an active opposable thumb increases hand articulations to enable more dexterous grasping.

## Main

**Fig. 1:**
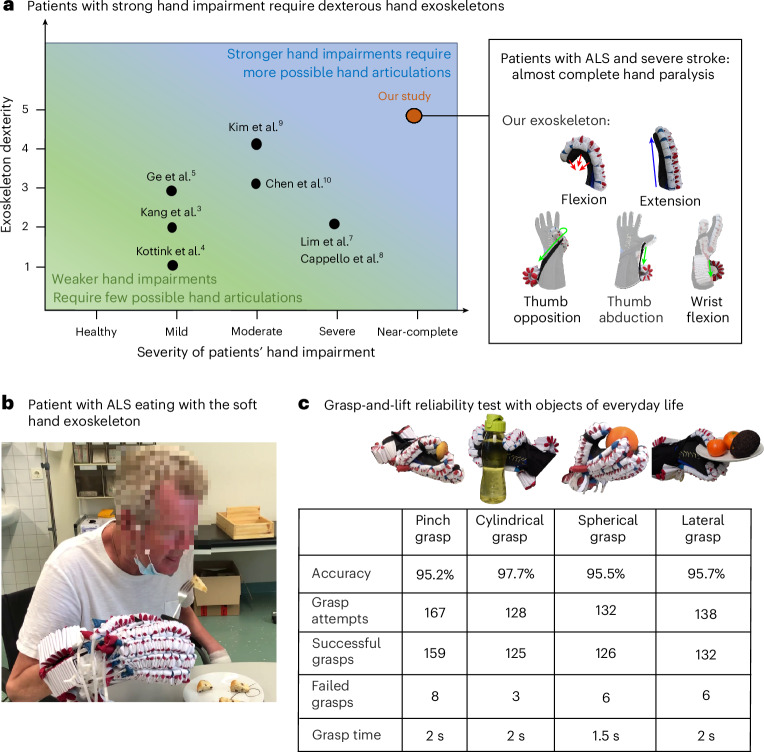
Dexterous soft hand exoskeleton to assist severely impaired hand function. **a**, Due to the severe and progressive impairment level of our patient’s hand function, we increased the exoskeleton dexterity by adding additional hand articulations compared with previous soft hand exoskeletons (thumb opposition, thumb abduction and wrist flexion) through iterative prototypes (P1, P2 and P3). For example, Kottink et al.^4^ use cable-driven actuation to provide finger flexion (one articulation, patients with medium hand impairment); Lim et al. and Cappello et al.^7,8^ use pneumatics to power two bidirectional flexor and extensor actuators (two articulations, patients with stroke and patients with C4–C7 spinal cord injury); Kim et al.^9^ use cable-driven actuation to provide finger flexion, passive extension and thumb opposition (three articulations, patients with stroke). Kang et al.^3^ use cable-driven actuation for finger flexion and extension; the thumb was supported only by an opposition cable mounted on a passive structure (three articulations, patients with spinal cord injury). Ge et al.^5^ use pneumatics actuation to support finger flexion and extension in addition to thumb abduction (three articulations, patient with brachial plexus injury). **b**, Using the soft hand exoskeleton, our patient was able to grasp a fork to feed himself. **c**, The dexterous hand exoskeleton enables the grasping of representative everyday objects with diverse shapes and functionally relevant weights. To evaluate the reliability, two healthy participants performed 10-min continuous grasp-and-lift experiments for each grasp type using a silicon hand model inside the exoskeleton.

Hand paresis is a frequent consequence of neurological disorders, including the motor neuron disease amyotrophic lateral sclerosis (ALS). Affecting approximately 5 people per 100,000 population^1^, ALS leads to progressive loss of muscle function, often impairing a patient’s ability to walk and grasp in the early stages of the disease. While wheelchairs are commonly used to assist with mobility, there is no widely accessible equivalent to support hand function. Recently, designs of soft hand exoskeletons have been proposed as a potential solution^2–10^. However, severely impaired patients, who are unable to contribute to hand preshaping before grasping, require more dexterous exoskeletons with a greater number of hand articulations to successfully pick up everyday life objects of various shapes such as cutlery and bottles. In particular, an active opposable thumb is necessary to perform a variety of grasp types such as a precision or power grasp. Unlike rigid exoskeletons, soft exoskeletons are lightweight and inherently safe, and can be tailored from inexpensive materials to fit an individual^11^. Soft robotic hand exoskeletons have made great strides in recent years, offering lightweight, textile-based solutions that support patients with limited hand function. However, most existing devices are still limited to simple finger flexion and extension with only partial thumb or wrist support and are typically tested on patients with moderate residual function. Supplementary Table 1 provides a comparative overview of recent soft robotic glove studies, highlighting differences in actuation type, control interface and target user population. Most of the state-of-the-art hand exoskeletons have been developed for patients with mild or moderate levels of hand impairment (Fig. 1a) and might not apply to patients with severe hand impairments or even hand paralysis. As a result, people with severe paralysis, such as patients with advanced ALS or severe stroke, often remain without effective available options.

**Fig. 2:**
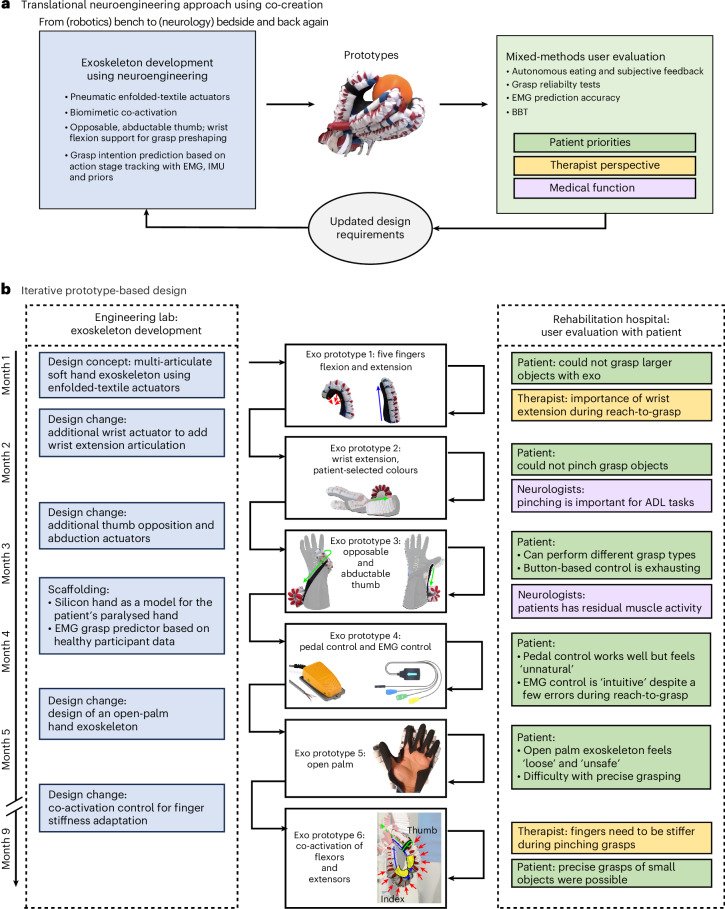
Co-creation approach. **a**, The translational neuroengineering approach utilizes exoskeleton prototypes for a mixed-methods evaluation together with the patient and medical professionals. The multiperspective feedback is translated into design improvement requirements, which are themselves translated into new exoskeleton designs using various neuroengineering methods. **b**, Time course of the co-creation process, presenting the sequence of six exoskeleton prototypes that were evaluated with the patient. Feedback and ideas from the patient (green), therapists (yellow) and neurologists (violet) were used as the basis for design changes. The successive addition of additional hand articulations was required owing to the patient’s severe and progressive hand impairment.

To address these challenges and enhance both usability and user acceptance, we co-created a high-dexterity soft hand exoskeleton in collaboration with a patient experiencing near-complete right-hand paralysis due to ALS. This process involved eight co-design sessions over a 9-month period, following an iterative translational approach of ‘from bench to bedside and back again’^12^. In contrast to traditional top-down development of assistive technologies, co-creation enables direct integration of user priorities and multidisciplinary clinical input through iterative prototype refinement^13^. Through mixed-methods prototype evaluation and repeated discussions with the patient, neurologists and physiotherapists, we identified core needs and design requirements specific to severe impairment and translated them into neuroengineering solutions combining methods from neuroscience and robotics^14–16^. In particular, the resulting design incorporated bio-inspired principles of human grasping, including hand preshaping, thumb opposition for contact-point selection, and stiffness modulation via co-contraction. These principles, together with error monitoring and correction during motor control, guided the development and refinement of successive exoskeleton versions throughout the co-creation process (Fig. 2). Following this co-creation phase, we validated the assistive performance and usability of our dexterous soft hand exoskeleton with six individuals who had hand function impairments due to a middle cerebral artery stroke. In particular, we investigated the difference in hand function improvement due to exoskeleton assistance between patients with severe hand impairment and patients with moderate hand impairment. The co-creation process was intentionally centred on a single individual with near-complete ALS-related hand paralysis to address the design requirements of severe impairment, while participants with other neurological conditions were included at later stages for validation and comparative analysis rather than to inform design iterations.

An iterative design process guided by the co-creation participant’s priorities of comfort, safety and independence resulted in a dexterous soft hand exoskeleton tailored to severe hand impairment. Rather than augmenting residual motor function, the system was explicitly designed to restore intentional grasping in individuals who cannot voluntarily preshape or stabilize the hand. In this work, we explicitly target individuals with severe to near-complete hand paralysis, for whom preshaping and grasp stabilization cannot be achieved voluntarily, and we contrast their outcomes with those of patients with moderate residual function to delineate the boundaries of assistive benefit. This impairment-specific design philosophy informed both the mechanical architecture and the control strategy, and distinguishes the present work from prior soft hand exoskeletons developed primarily for moderate impairment. The following sections describe how co-creation translated patient needs into engineering requirements, and how the resulting system was evaluated across different impairment levels.

## Results

### Co-creation reveals patient priorities and engineering requirements for assisting individuals with severe hand impairments

Through guided discussions and prototype testing, we identified the patient’s goals and preferences regarding how the hand exoskeleton should support him (Fig. 2). He expressed a strong desire to eat independently and emphasized that comfort, feeling in control, safety and reliability were his top priorities. In addition, we evaluated the patient’s ALS-related challenges and residual abilities through medical assessments and consultations with a physiotherapist. Without the exoskeleton assistance, he could not move any finger of his right hand, except for small movements with the distal phalanx of his right thumb. With the initial prototype of our soft hand exoskeleton that exclusively supported finger flexion and extension, he was unable to grasp objects of diverse shapes. In contrast to less severely impaired individuals, his impairments extended beyond insufficient grip force, which traditional flexion–extension exoskeletons can assist with. In addition, his individual assistive needs included supporting the fine motor coordination of the fingers during the grasping process. Addressing this challenge, a physiotherapist emphasized the importance of preshaping the hand before grasping, particularly through thumb opposition and abduction, and extending the wrist to facilitate hand opening and enable proper orientation during reach-to-grasp movements. Drawing from these insights, we derived key engineering design requirements, namely that the exoskeleton should be soft and lightweight, hands-free and intention-driven (for example, via residual myoelectric activity) and capable of supporting the grasp of objects with varying shapes and weights. In addition to grip strength assistance, our exoskeleton ought to leverage bio-inspired principles of dexterous hand function to support finger coordination for reliable and natural grasps.

### Thumb dexterity enables reliable grasping

**Fig. 3:**
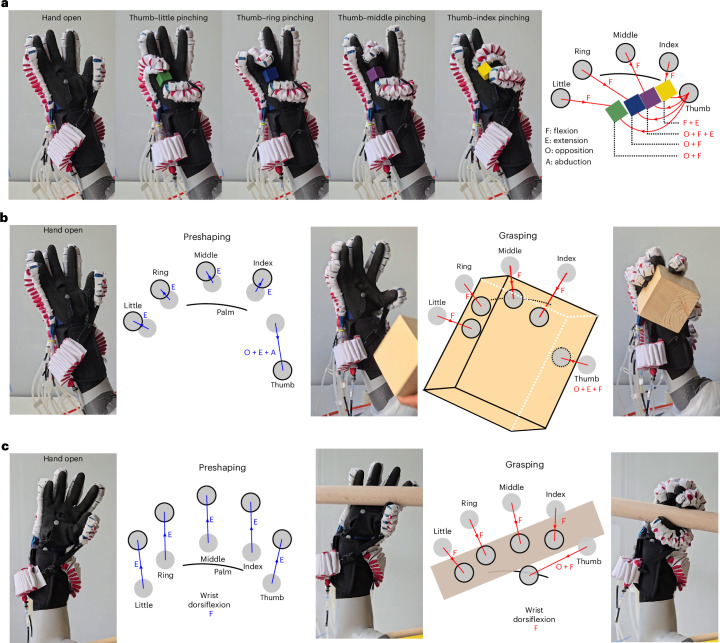
The hand exoskeleton enables high dexterity: exoskeleton pictures and simplified illustrations on preshaping and grasping. **a**, Pinching is possible with each of the four fingers and the thumb. The hand exoskeleton enables high dexterity in pinching by coordinating various thumb actuators, including extension, flexion and opposition. Thumb–index pinching is achieved through index finger flexion combined with thumb flexion and extension. Thumb–middle pinching involves middle finger flexion and thumb flexion, extension and opposition. Thumb–ring and thumb–little pinching are enabled by the respective finger’s flexion along with thumb flexion and opposition. **b**, Hand preshaping before grasping enables the fingers to come into contact with three surfaces of the large cube (7.5 cm) to successfully grasp it. **c**, Hand preshaping with wrist dorsiflexion increases the contact area between the palm and the wooden stick (100 cm, 1 kg), enabling support against gravity and allowing a successful grasp through finger flexion and thumb opposition, as the fingers enclose the object.

The thumb plays a central role in grasping, contributing to over 40% of total hand functionality^17^. Due to its ability to perform flexion, extension, abduction and opposition, the thumb can align and press effectively against any of the four fingers, enabling both stable pinch and power grips. Our bio-inspired actuation system equipped with an active opposable and abductable thumb replicates the natural coordination of these thumb movements and enables our exoskeleton to successfully perform the finger-to-thumb opposition task, which includes sequentially pinching between the thumb and each finger (Fig. 3a). This ability to perform thumb opposition, the movement of the thumb across the palm towards the fingers, is critical for forming multipoint contact during grasping. This enhances grip stability and distributes force, reducing the likelihood of object slippage. Thumb abduction, which moves the thumb away from the palm, expands the functional workspace for larger objects (Fig. 3b) and facilitates the initiation and adjustment of grasp configurations, particularly for wide or irregularly shaped objects. With all four fingers capable of flexion and extension, the soft hand exoskeleton can adaptively wrap around objects of varying shapes and sizes. The dexterous thumb complements this by positioning itself to generate an opposing force, which is essential for grasp closure and load support (Fig. 3c). Together, these coordinated thumb motions enable the exoskeleton to perform a wide range of grasp types, improving its reliability across diverse tasks from manipulating small tools to securely holding larger items such as bottles.

To evaluate the exoskeleton’s reliability, two healthy persons each performed 10-min continuous grasp-and-lift actions for each grasp type using a passive silicon hand inside the exoskeleton glove as a model of the patient’s paralysed hand. For each action, one out of four everyday life objects (bottle, pastry, orange and bowl) needed to be grasped with a fitting grasp type, lifted and transported a distance of 30 cm to a target square (10 cm). For all grasp types, more than 125 successful grasp-and-lift actions could be achieved with >95% reliability. The total number of grasp attempts, successful grasps and failed grasps are displayed in Fig. 1c.

### Hand preshaping enables contact point selection

Effective grasping begins with appropriate hand preshaping by configuring the fingers and thumb before contact to optimize grasp stability. Our soft hand exoskeleton achieves this through coordinated control of the wrist dorsiflexion actuator, fingers’ extension actuators and thumb abduction actuator. As illustrated in Fig. 3c, wrist dorsiflexion orients the hand to better align with the object’s geometry and its anticipated centre of mass. The extension actuators position the fingers in an open, ready-to-grasp posture, while thumb abduction increases lateral spread, enlarging the grasp aperture (Fig. 3b). Individual finger actuation allows fine-tuned adjustment of contact points to conform to the object’s surface. This preshaping strategy addresses two key objectives: (1) counteracting gravitational torque caused by the object’s centre of mass to enhance grasp stability, and (2) maximizing frictional forces by aligning finger surfaces to maximize normal contact force vectors. These coordinated motions allow the hand to establish a stable and secure grasp before object contact, increasing overall grasp reliability across a range of object shapes, weights and orientations^18^.

### Co-creation enables customization for severe impairment

**Fig. 4:**
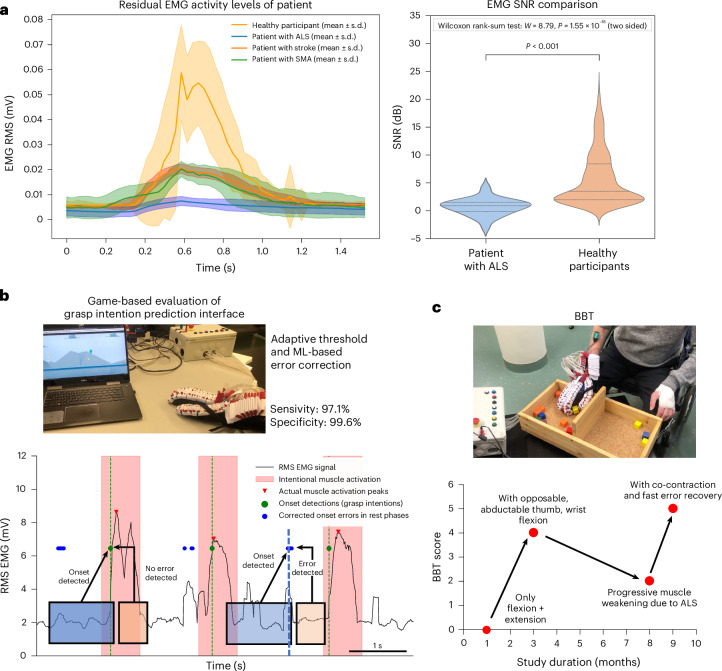
Intentional control of exoskeleton grasping using the patient’s residual EMG activity. **a**, Left: averaged peak-aligned time-course of EMG-RMS muscle activations of the patient with ALS P1, patient with stroke P6 and patient with SMA P7, compared with a healthy participant. Right: the SNR of the patient’s EMG signal during intentional muscle activation was significantly lower compared with healthy participants (*n* = 15) using the non-parametric Wilcoxon rank-sum test (*W* = 8.79, *P* = 1.55 × 10^−18^, two sided). **b**, Performance of real-time EMG-based grasp intention prediction during the game-based evaluation experiment with the patient with ALS. Using the adaptive threshold onset detection and ML-based error correction trained on his EMG data, the system correctly detected 97.1% of his grasp intentions that were cued by the game. **c**, The initial flexion–extension exoskeleton version did not increase the patient’s BBT score; however, design changes made through our co-creation process to adapt the exoskeleton dexterity to his severe hand impairment level increased it to a BBT score of 5, despite progressive muscle weakening.

As shown in Fig. 2b, the co-creation sessions with the patient resulted in multiple iterative designs of our exoskeleton. Based on his feedback, our observations and input from therapists and clinicians, we added additional degrees of freedom such as active thumb opposition, hand preshaping and a more intuitive electromyography (EMG)-based control interface. We retained a closed-palm design for the exoskeleton glove, which he preferred over an open-hand design due to its firm fit, reflecting his prioritization of safety. Developing soft hand exoskeletons with textile material enables customization to the hand size and shape of individual patients, thus further contributing to a stable and robust attachment. With the initial exoskeleton prototype based on finger flexion and extension, the patient with ALS could only grasp medium-size objects using a power grasp without opposed thumb and scored 0 on the Box-and-Blocks Test (BBT) (Fig. 4c, at month 1). By contrast, after the co-creation process, using the final exoskeleton design with additional actuators for thumb opposition, abduction and wrist dorsiflexion, as well as bio-inspired approaches to hand preshaping, co-activation and error correction, he was able to score 5 on the BBT (Fig. 4c, at month 9). In addition, the co-created design changes enabled him to reliably grasp a bottle (cylindrical grasp), a plate (lateral grasp), an apple (spherical grasp) and a pastry (pinch grasp), thus demonstrating the capability of the hand exoskeleton to support multiple grasp types for representative everyday objects relevant to daily living tasks. The hand preshaping before each grasp using the thumb abduction, opposition and wrist flexion actuators allowed the patient to optimally position his hand for stable grasps. Thus, the co-created exoskeleton substantially improved his hand function compared with the initial design. Critically, and most important to him, the exoskeleton enabled the patient to feed himself independently (Fig. 1b).

### Validation of hand function assistance and usability with patients with stroke

**Fig. 5:**
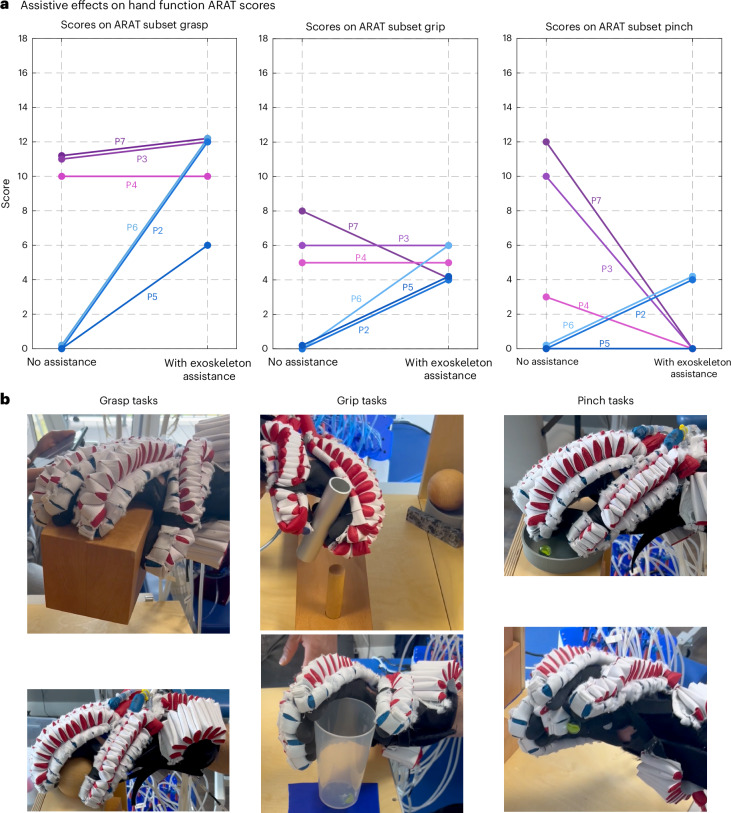
Validation with patients with stroke. **a**, When assisted by the soft hand exoskeletons, patients with stroke with severe hand impairment (P2, P5 and P6—shown in shades of blue) achieved significant improvements in the ARAT. By contrast, the hand function of patients with stroke with moderately strong hand impairments (P3, P4 and P7—shown in shades of violet) improved only for the grasp subtask involving grasping a large and heavy object, but did not improve for the other tasks and was reduced for pinching tasks. **b**, Participants performing ARAT hand function tasks while assisted by the soft hand exoskeleton.

After finalizing the hand exoskeleton design through the co-creation process with our patient with ALS, we validated its assistive performance on six individuals with hand impairment due to a recent stroke. All patients with stroke had suffered an ischaemic infarct in their middle cerebral artery that resulted in hemiparesis and were in early neurorehabilitation. The patients were selected to fit into two groups of three individuals, each based on their non-assisted performance on the BBT. The three patients who were unable to grasp and lift a single block and therefore obtained a BBT score of 0 were classified as the heavily impaired group, whereas the other three patients with stroke, with BBT scores of 19, 6 and 12, were classified as the moderately impaired group. These experiments were designed to assess assistive performance across impairment levels and did not inform the exoskeleton’s mechanical or control design, which was finalized during the preceding co-creation phase. Importantly, this stratification was intentional, as the exoskeleton was not designed to augment fine motor control in patients with moderate residual function, but rather to restore basic grasping capability in those with severe paralysis. To evaluate the level of functional assistance that the exoskeleton provided to these patients, we asked them to perform a modified version of the Action Research Arm Test (ARAT)^19^ twice—once without the hand exoskeleton and once with the assistance of the hand exoskeleton. Because we were exclusively interested in their hand function but not in their arm or shoulder function, we manually supported the patient’s arm movements during the tasks. The ARAT test consists of four subsets: grasp (see example tasks in Fig. 5b, left), grip (see example tasks in Fig. 5b, middle), pinch (see example tasks in Fig. 5b, right) and gross movement. Due to our focus on hand function, we did not assess the gross movement subset. Validation focused on standardized clinical assessments of hand function rather than task-specific daily living activities. Repeatability of preshaping and grasp execution across multiple repetitions and ARAT-relevant objects, evaluated using a passive silicone hand model to isolate device behaviour, is illustrated in Supplementary Video 13, including a water-pouring task that demonstrates stable grasping during lifting, wrist reorientation and dynamic load changes. Each task was repeated for ten trials, with successful grasp execution achieved in all repetitions. Task completion times were consistent across repetitions, with average execution times of 7.87 ± 1.12s (water pouring), 5.42 ± 0.40 s (10-cm block), 5.05 ± 0.77 s (stone), 1.99 ± 0.23 s (2.5-cm block), 2.87 ± 0.29 s (cricket ball), 5.52 ± 0.89 s (1-cm tube) and 4.63 ± 0.47 s (2.25-cm tube). These reported execution times were computed from the predefined motor command sequences. The start of each trial was defined as the instant corresponding to the first actuator activation (*t* = 0), triggered by the operator of the dummy arm, while the end time was determined by the completion of the final release phase, also initiated by the operator via the control interface. This introduces a limited user-dependent component in the timing, associated with the initiation and termination of the sequence. However, once triggered, the execution follows a deterministic motor program with millisecond resolution, ensuring consistency across repetitions. The measured durations therefore reflect the combined effect of the programmed coordination of actuator commands, the intrinsic pneumatic response of the system, including valve dynamics and pressure evolution, and the operator’s interaction during task execution.

Our results are presented in Fig. 5a and show that the hand exoskeleton assistance led to an improvement in hand function for the patients with stroke in the severely impaired group in the grasp subset (average improvement of 10 points) and grip subset (average improvement of 4.67 points). For the moderately impaired patients, using the exoskeleton only led to a score improvement for P3 and P7 by one point each as it enabled both patients to grasp and robustly lift the large and heavy cube (edge length 10 cm), which slipped out of their hands without exoskeleton assistance owing to their limited grip force. When they were assisted by the exoskeleton, their combined grasp force was strong enough to robustly hold the object against gravity. In the grip subtask, exoskeleton assistance did not improve the performance of the moderately impaired patients, with P3 and P4 obtaining equal scores with and without the exoskeleton, while the score for P7 reduced when being assisted. We also observed a reduction of hand function scores in the pinch subtask, albeit only for the patients in the moderately impaired group. For two of the severely impaired patients, we observed a slight increase in their score when assisted by the hand exoskeleton, as they were able to grasp and lift the glass marble between their index finger and thumb and also between their middle finger and thumb. We hypothesize that this score improvement for severely but not moderately impaired patients was due to motivational differences caused by the sequence of tasks. The severely impaired patients attempted the pinch tasks directly after having experienced a large functional improvement due to the exoskeleton support and therefore had a higher expectation and motivation to achieve the task goals. These findings suggest that the assistive strategy is most effective when voluntary preshaping and grasp stabilization are absent, and may be less suitable when residual motor control is preserved.

In addition to the functional assessments, we obtained user feedback from all six patients with stroke on the usability of the exoskeleton by administering the System Usability Scale (SUS) questionnaire^20^. The SUS is a well-established and validated tool for assessing usability. It includes ten statements (or ‘items’) that respondents rate on a 5-point Likert scale, ranging from 1 (strongly disagree) to 5 (strongly agree). Over all six patients with stroke, we obtained an average SUS score of 65.4 out of 100 (standard deviation (s.d.) 18.6), which according to ref. ^21^ corresponds to an ‘OK’ to ‘Good’ usability. This is similar to the average SUS scores reported for other, differently actuated hand exoskeletons (mean SUS score of 60.6 for the TenoExo in ref. ^22^), which was evaluated in patients with spinal cord injury with varying levels of impairment more comparable to our moderately impaired group. We observed a higher mean SUS score for the severely impaired patients (mean 68.3, s.d. 17.7) than for the moderately impaired patients (mean 62.5, s.d. 22.9). These results suggest that exoskeleton usability might be affected by the individual level of impairment; however, due to the small sample size, no statistical effect could be observed.

### Grasping intention can be detected from non-invasive EMG signals despite low SNR

**Fig. 6:**
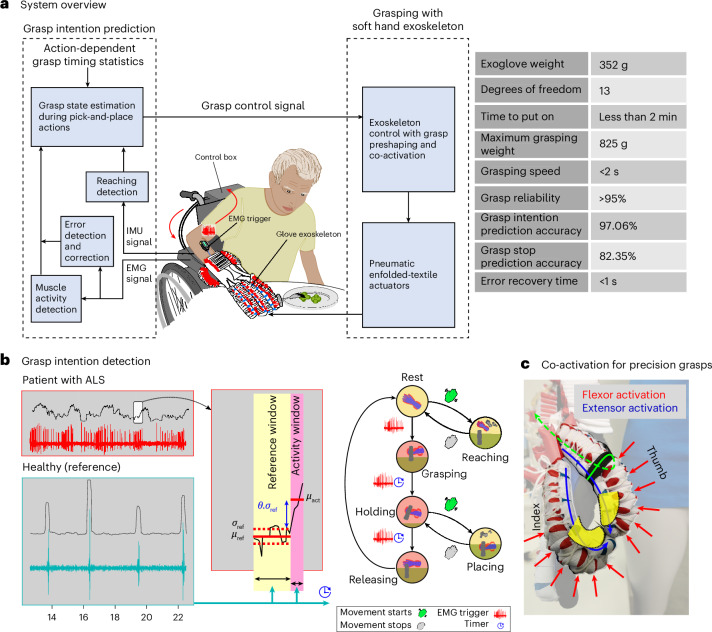
System overview and grasp intention detection. **a**, The patient’s grasp intention (categorized as close hand, hold grasp and open hand) is detected using non-invasive sEMG and IMU signals from his forearm and sent as a control signal to the soft hand exoskeleton, which moves his fingers accordingly to enable grasping. **b**, We predicted the patient’s grasp by detecting increases in muscle activity (sEMG) in his residual flexor pollicis longus using an adaptive threshold algorithm. In comparison with healthy participants, the patient’s EMG signal is weaker and noisier (Fig. 4a). To avoid misclassifications due to motion artefacts during reaching, we use motion sensor data (IMU) to estimate the state during pick-and-place actions, which is used as contextual information for robust intention prediction. **c**, Simultaneous co-activation of flexors and extensors increases finger stiffness and enables precise grasping of small objects.

The patient with ALS preferred a surface EMG (sEMG)-based intentional control interface over button-based control because it allowed him to control the exoskeleton in a hands-free manner with low delay, more natural than pressing a button. Control was achieved by contracting the flexor pollicis longus, a muscle normally involved in grasping and the only muscle in his right hand over which he still retained residual voluntary control. However, his EMG signal was noisy and weak compared with that of a group of healthy participants (*n* = 15) and also visibly smaller than the average activation peaks of one of the patients with stroke and a patient with spinal muscular atrophy (SMA) (Fig. 4a left), making robust intention prediction challenging. Previous research by Meier et al.^23^ has pointed out this challenge of reliable EMG onset detection for individuals with hand impairment, reporting an accuracy 95.4% for healthy participants and a range of 47.0–91.6% for patients with different levels of impairment. Using the non-parametric Wilcoxon rank-sum test, our results showed that the signal-to-noise ratio (SNR) of the patient’s EMG signal over 50 intentional muscle contractions of his flexor pollicis longus was significantly lower (*P* < 0.001, *z* = 8.79) than of a group of healthy controls (*n* = 15, in a total of 1,213 muscle activations) (Fig. 4a, right). Using an adaptive thresholding method for muscle activity onset detection (Fig. 6b), the EMG-based system detects the user’s grasp intention to enable control of the soft hand exoskeleton (Fig. 6a). To train the patient to use this intentional control interface, a game-based testing paradigm was created (Unity3D) in which he was given multiple consecutive action cues at specific non-regular timepoints. When a cue was given, he needed to contract his muscle within a short time window (<0.5 s). If the system recognized his EMG activity onset within this time window, the trial was counted as correct and visual feedback was given in the game. The highest task performances (percentage of correct trials) were achieved in the start-grasping condition (average 97.1%). In the stop-grasping condition, when the exoskeleton assisted grasping and the hand was closed, the intentional activity could also be detected (average 82.4%). Task performance was higher when a high sensitivity of the model was chosen (Fig. 4b).

In addition to this real-time evaluation, we compared machine learning (ML)-based onset detection approaches trained on three different EMG-root mean square (RMS) datasets (healthy, healthy plus SMA, and ALS) and evaluated on the EMG activity of the patient with ALS where he was asked to repeatedly perform intentional muscle activations of his flexor pollicis longus as if wanting to trigger an exoskeleton grasp. When training a one-dimensional (1D) convolutional neural network (CNN) model on the flexor pollicis longus EMG-RMS data of 15 healthy participants, and evaluating it on the ALS EMG-RMS data, we obtained a sensitivity of 100% and a specificity of 90.6%, that is, the model could detect grasping intentions accurately but resulted in a large number of false positives. Adding flexor pollicis longus EMG-RMS data from an individual with SMA and retraining the model retained the high sensitivity, and the specificity increased sightly to 90.9%. We obtained the best results when the model was trained exclusively on data from the patient with ALS (on a training dataset separate from the test dataset), which resulted in 100% sensitivity and 96.7% specificity, suggesting a personalization effect of the model training. Notably, the retention of high sensitivity when training on data from healthy participants and evaluating on EMG from the patient with ALS, despite differences in recording sessions, electrode placement and neuromuscular pathology, suggests a degree of cross-session and cross-participant robustness in the learned onset representations. This is consistent with recent findings that EMG decoding models can generalize across users and sessions when trained on sufficiently large datasets^24^. Furthermore, advances in unsupervised online adaptation^25^ offer promising strategies to further reduce recalibration burden in future system iterations, particularly for progressive conditions such as ALS where signal characteristics change over time. A Grad-CAM analysis showed that the trained onset detection model focuses on regions of increasing and high EMG-RMS signal in the latter portion of the detection window, consistent with the temporal characteristics of physiological muscle activity onsets. However, for our co-creation sessions, the adaptive threshold approach proved more applicable than the ML approach as it allowed us to quickly adapt the sensitivity to the patient’s daily condition without requiring to record data and retrain the model. In addition, it detected grasp intentions earlier than our CNN models, with a mean of 201 ms before the muscle activity peaks compared with 5 ms. The resulting responsiveness of the exoskeleton grasp triggering was important to the patient.

To quantitatively assess the patient’s ability to pick-and-place objects, we performed the BBT^26^ in several sessions spread over the duration of the study. Due to the paralysis of the patient’s right hand, he could not transfer any blocks without the assistance of the hand exoskeleton, leading to a floor effect on this clinical ability test. Wearing the final version of the hand exoskeleton and using the intentional control interface, he was able to transfer up to five blocks within 60 s with minimal assistance (Fig. 4c). For the co-creation participant, achieving a non-zero BBT score corresponded to the ability to intentionally grasp, transport and release objects, enabling independent feeding. During the course of this study, the progression of his disease resulted in a lower BBT performance, which could be counteracted by adapting the exoskeleton design, for example, by increasing finger stiffness during pinching through flexor–extensor co-actuation.

### Error monitoring and correction enables fast recovery

Our approach, which uses an adaptive threshold with high sensitivity for muscle activity onset detection, had the advantage that grasping intentions of the patient with ALS were predicted reliably and early. This was important to him as it supported his feeling of control over the exoskeleton. Subjectively, for him a high sensitivity was preferred to high specificity, stating ‘When I want to grasp, it should understand that and help and not do nothing.’ (translated from German). However, this came with the drawback of a higher false-positive rate, leading to the exoskeleton grasps being triggered during rest periods (94.6% specificity). Inspired by error monitoring processes in human motor control^27^ and real-time error detection and correction approaches in brain–machine interfaces^28,29^, we trained a CNN on EMG data from the patient with ALS to determine after each onset detection whether the onset was erroneously caused by non-intentional signal variability during a resting phase or correctly reflected the start of intentional muscle activity. Using this post-hoc error detection enables us to stop the incorrect exoskeleton grasp action within 500 ms after the trigger is being sent. On ALS EMG test data, this approach enabled us to detect most false-positive onset detections during resting phases, increasing specificity to 99.6% (representing a 13.5-fold reduction of the error rate). None of the true-positive onset detections was erroneously corrected, therefore leading to no reduction in sensitivity and, thus, grasp intention prediction accuracy. This large increase in specificity was possible because the error-detection model was trained with a larger detection window and analysed data later during intentional muscle activation. We suggest that, similar to error monitoring in humans, this two-process approach of combining fast, error-prone models with slow but accurate models is a promising solution to the responsiveness–accuracy trade-off in brain–machine interfaces.

## Discussion

In this study, we demonstrate that restoring hand dexterity through multi-articulate soft hand exoskeleton and impairment-adapted control can enable meaningful object manipulation in individuals with severe hand impairment, even when myoelectric signals are severely degraded by neuromuscular disease.

A key finding of this study is that the utility of assistive hand exoskeletons is strongly dependent on the level of residual hand function. While patients with severe impairments benefitted substantially from the additional dexterity and preshaping support, some moderately impaired patients experienced no benefit or even reduced performance. This highlights that exoskeletons optimized for severe paralysis may interfere with voluntary motor strategies in patients with higher residual function, underscoring the need for impairment-specific assistive designs.

The limited or negative performance changes observed in moderately impaired patients with stroke probably reflect a combination of factors rather than a failure of the assistive approach. Notably, the present design does not explicitly address spasticity or abnormal muscle tone, which are common after stroke and may require distinct mechanical compliance, control strategies or therapeutic integration. First, the exoskeleton was explicitly designed to restore grasping capability in individuals who cannot preshape or stabilize the hand voluntarily, and therefore its level of assistance and coordinated actuation may interfere with intact or partially intact motor strategies in patients with moderate residual function. Second, several ARAT tasks, particularly in the grip and pinch subsets, require fine motor coordination and adaptive force modulation, which may be constrained by the exoskeleton’s predefined grasp patterns and compliance characteristics. Third, the intentional control strategy was optimized for users with minimal residual activity and prioritizes robustness and responsiveness over fine-grained modulation, which may limit its suitability for patients who can already initiate and modulate grasp movements voluntarily. Together, these factors suggest that the observed results primarily reflect a mismatch between device design and impairment level, while also highlighting task- and control-specific limitations that should be addressed in future impairment-adaptive exoskeleton systems.

In current clinical practice, assistive options for impaired hand function primarily include passive orthoses, static or dynamic splints, and simple active devices that provide uniform finger flexion or extension, often controlled via buttons or switches. While such systems are comparatively robust and easier to deploy, their limited dexterity and lack of coordinated thumb opposition or preshaping support restrict their effectiveness, particularly for individuals with severe to near-complete hand paralysis who cannot voluntarily configure the hand before grasping. In this context, the proposed system does not seek to replace existing standards of care, but rather to address a functional gap for patients who derive limited benefit from less dexterous devices, demonstrating how increased articulation and adaptive control can restore intentional object manipulation when simpler assistive strategies are insufficient. The clinical meaning of changes in standard outcome measures such as the BBT and the ARAT depends strongly on the baseline level of impairment. For individuals with severe to near-complete hand paralysis, these assessments exhibit pronounced floor effects, such that non-zero scores reflect the emergence of a qualitatively new functional capability rather than incremental improvement. In this context, the ability to transfer any blocks in the BBT or to complete grasp and grip items in the ARAT corresponds to regaining intentional object manipulation, which directly translates to activities of daily living such as holding utensils, stabilizing objects or feeding. Thus, although absolute score changes remain modest, they represent functionally meaningful transitions from inability to capability for severely impaired patients. While activities of daily living provide an important motivational context, the present study evaluates assistive benefit primarily through standardized clinical measures and functional grasping tasks, rather than comprehensive ADL performance.

Within a translational framework, the single-patient co-creation study should be interpreted as both exploratory and demonstrative. The presented system is highly individualized, and key design parameters, including actuator configuration, thumb geometry, open or closed palm, intentional control approach and EMG electrode placement, were tailored to a single participant and are not expected to transfer directly to other patients without substantial adaptation. Nevertheless, as they emerged as design priorities from a structured co-design process grounded in clinical and user evaluation, they offer a documented rationale that may guide personalization for other patients. The iterative design process, bio-inspired actuation strategies and intentional control interface were explored and refined exclusively in close collaboration with one individual with near-complete ALS-related hand paralysis, and therefore primarily demonstrate feasibility rather than population-level efficacy. At the same time, the resulting ability to reliably perform diverse grasp types and to regain a meaningful activity of daily living, namely independent feeding, serves as a demonstrative proof of principle for what multi-articulate soft hand exoskeletons can enable when tailored to severe impairment. Findings related to control performance, usability and functional gains should not yet be generalized beyond this impairment level or design configuration, motivating the subsequent validation with patients with stroke and future studies with larger cohorts.

Several limitations related to scalability and deployment remain and frame the current translational gap of the presented system. First, the high level of dexterity required to assist individuals with severe to near-complete hand paralysis results in increased system complexity, including multiple pneumatic actuators, valves, sensors and control components, which currently limits ease of set-up and maintenance. Second, pneumatic actuation, while lightweight, compliant and well suited for soft wearable devices, requires an integrated air supply. In the current system, this results in a compressor–valve–electronics unit weighing approximately 2 kg, which constitutes a non-trivial limitation in terms of portability. While the glove itself is lightweight, the overall system currently restricts fully independent use and represents a significant barrier to deployment in unsupervised home environments. Reducing the size, weight and power requirements of this control unit is therefore a key requirement for future translation. Third, the intentional control interface requires individual calibration and periodic adjustment to accommodate day-to-day variability in residual muscle activity. Particularly in progressive conditions such as ALS, longitudinal repeatability is of high clinical relevance. While threshold adjustments in combination with error monitoring and correction can help to counteract disease-related EMG signal reduction, developing and evaluating methods for improving cross-session reliability, particularly over long time horizons, remains an important open research question Together, these factors indicate that, while the system demonstrates translational feasibility and clinical relevance, further engineering efforts are required to reduce hardware complexity, automate calibration and further improve robustness before deployment outside supervised clinical environments.

A major contributor to successfully re-enabling the patient to grasp diverse objects was our translational co-creation approach, where multiple prototypes of the hand exoskeleton were evaluated together with the patient, and his feedback was incorporated into the subsequent exoskeleton design changes. Our co-creation process exemplifies the value of moving beyond the user-centred design approach in assistive robotics where largely passive users are observed and interviewed, towards a participatory development process where users are actively testing multiple prototypes^13,30^, enabling them to give tangible and concrete feedback on their experiences and priorities. However, within healthcare contexts, co-creation approaches can place a burden on patients regarding time commitment and can lead to exhaustion. To prevent this, we added scaffolding to our co-creation process, by building a silicon hand model that we could place inside the hand exoskeleton to simulate his paralysed hand. This allowed us to perform high-frequency functional tests with new prototypes before our evaluation meetings with the patient. A fundamental limitation of co-creation studies involving a single participant is the generalizability of findings. Therefore, we validated the assistive function of the co-created exoskeleton with several patients with stroke who had different levels of hand impairment. On average, the severely impaired patients reported higher levels of exoskeleton usability than the moderately impaired patients. However, the large variability between patients, including within the two groups, indicates that there are additional factors influencing the SUS scores.

Moving towards clinical translation, several next steps are required. These include larger, controlled studies to evaluate functional outcomes and usability across a broader range of patients with severe hand impairments, as well as investigations to assess robustness and adaptation over time, particularly in progressive conditions such as ALS. Future evaluations should also quantify user cognitive load and control intuitiveness using established dual-task paradigms. In parallel, regulatory considerations, safety certification and integration into existing clinical workflows will be essential to enable deployment beyond research settings. Together, these steps outline a clear pathway from the present proof-of-principle towards clinical and home use. Future studies should systematically evaluate performance across broader object weight ranges to quantify load limits and generalization to diverse daily tasks.

In conclusion, we present a co-created, multi-articulate soft hand exoskeleton designed specifically for individuals with severe to near-complete hand paralysis, demonstrating that restoring dexterity, rather than merely augmenting strength, is critical for this adult patient population. Adapting the hand exoskeleton to the patient’s individual requirements and high impairment level required a higher number of exoskeleton articulations that go beyond strength assistance during flexion and extension but also support more dexterous hand movements that involve thumb opposition, abduction and wrist flexion. The increased exoskeleton dexterity furthermore enabled the implementation of biomimetic grasping strategies such as hand preshaping to increase hand aperture during reach-to-grasp, to increase hand–object contact friction and to enable contact point selection for stable grasps.

Together, the validation results reinforce that assistive benefit and usability are strongly dependent on impairment severity, underscoring the importance of matching exoskeleton design to residual motor function. In general, this requirement for adaptability of exoskeleton articulations to the individual impairment levels of patients and potentially to their progressive functional decline due to neurodegeneration (for example, with our patient with ALS) fits well with the flexibility and customizability that the soft robotics approach to exoskeleton design offers. Using clothing fabrics to construct an exoskeleton makes it lightweight and safe and enables tailoring the exoskeleton to a person’s size and according to their individual priorities and medical needs. Our findings show that co-created, multi-articulate soft exoskeletons restore dexterity beyond strength assistance, charting a scalable path for patient-specific neurorehabilitation. Bridging the remaining translational gap will require reducing system complexity, substantially reducing system size and weight to enable independent home use, and automating calibration to enable routine clinical and home deployment.

## Methods

### Patients

The patient participant in our co-creation approach was 65 years old and had been diagnosed with ALS 4 years earlier. The diagnosis was made on the basis of El Escorial World Federation of Neurology criteria, in accordance with the Gold Coast diagnostic criteria^31^, based on progressive distal weakness with atrophy of the small hand muscles and the thigh and calf muscles on both sides. The patient was treated with riluzol and edaravone^32^. At the time of the study, he presented with tetraparesis with distal weakness of all extremities. While the patient could move his arms in the shoulders and elbows against resistance (strength 4–4+/5 on the Medical Research Council scale), the muscles in both forearms and hands were almost limp with only minimal active movement of the right thumb (strength 1–2/5), no active finger movement of the right hand (strength 0/5) and severe paresis of the left hand (strength 1–2/5 throughout). There were clinically visible and EMG-confirmed fibrillations and fasciculations of the tongue, arms, legs and trunk. Reflexes were elevated as signs of upper motor neuron involvement. There were no sensory deficits, and coordination was normal. The patient could follow the discussions and instructions during the co-creation sessions without restriction and could communicate fluently. He declared informed consent before the start of the study, which was approved by the Ethics Board of the Technical University of Munich Hospital, Klinikum Rechts der Isar (167/21 S-EB). To avoid fatigue, the co-creation sessions were designed to last less than 60 min. The patient was selected due to the relative stability and initially minor neurodegeneration of his neurological condition which made it possible to work with him in our multiple months long co-creation study. When functional improvements were observed, we could attribute it with high certainty to the improvement in the exoskeleton design and control. By contrast, most patients with stroke are less suitable for longitudinal co-creation studies owing to their often rapid and sometimes spontaneous recovery, which makes causal attribution of functional improvement to either the assistive technology or their neurorehabilitation difficult.

The patient participants involved in the validation experiments were all undergoing early neurorehabilitation therapy after having experienced an ischaemic middle cerebral artery stroke several weeks before our experiments. Their ages spanned from 61 years to 83 years (average age 77.3 years). On average, patients in our severely impaired group were younger than in the moderately impaired group (74 years compared with 80.7 years). Their average chronicity (time interval between the stroke and inclusion to the study) was 11 weeks, with the patients in the severely impaired group being included on average after 12 weeks and the moderately impaired patients being included after 10 weeks on average. An overview of all participants is given in Supplementary Table 2. The participant with SMA did not perform exoskeleton-assisted tasks and was included exclusively to provide comparative EMG signal data for neuromuscular disease, enabling contextualization of signal quality and onset detection performance. The validation experiments were part of a study for which all patients provided informed consent before the start of the experiments, which was approved by the Ethics Board of the Technical University of Munich Hospital, Klinikum Rechts der Isar (2023-436_1-S-NP).

### Soft robotic hand exoskeleton

The lightweight (352 g) soft textile-based hand exoskeleton was built from ordinary clothing fabrics to make it comfortable to wear and is driven by 13 pneumatic Enfolded-Textile Actuators^33^ stitched into the fabrics of a hand glove structure. The flexor actuators are made from an inflatable textile tube corrugated through a housing textile structure (Fig. 1a). Inflating the tube produces pushing forces between the successive folds, bending the actuator and flexing the finger. Safe flexing forces are provided by limiting the air pressure to below 2 bar. The extensor actuators were designed as two parallel segments alongside the finger, which exclusively produce straightening forces. This design prevents hyperextension of the finger joints. Following pinch grasping tests with the patient, which showed that assistance of finger flexion and extension is insufficient when hand function is severely impaired, we added actuators for abduction and opposition to the thumb. The abduction actuator consists of a pulling ribbon attached from one side to an enfolded-textile actuator, and attached from the other side to the glove at the thumb’s metacarpophalangeal joint level. The enfolded-textile actuator is attached to the distal end of a sleeve worn on the lower forearm. Inflating the tube causes bending of the actuator and therefore pulls the ribbon, producing a thumb abduction. In a similar structure, the opposition actuator consists of a ribbon wrapped from one side around the thumb and routed over the ventral side of the wrist to the enfolded-textile actuator attached to the distal ulnar side of the sleeve. Inflating the tube produces a pulling force along with the ribbon, causing thumb opposition. The torque applied in thumb opposition will be more significant in the case of co-activation of the thumb flexor or extensor as the lever of the pulling force increases. During our first co-creation session, a therapist emphasized the importance of wrist dorsiflexion during reach-to-grasp motions. To support this, we added a wrist actuator extending the hand up to an angle of 35°, thereby enabling pre-grasp hand opening and allowing the participant to position his hand more easily about the objects he intended to grasp. This was important because his shoulder weakness precluded him from performing precise reaching movements.

The control box of the system houses a fully integrated air supply, including a compact compressor (860 g) and a manifold of 13 pneumatic modules. Each module combines a lightweight high-torque servo motor (19 g) with a three-way stopcock, a design specifically developed for applications with multiple textile tubes, such as the hand exoskeleton, where traditional two-valve-per-tube configurations would be bulky and expensive. The three-way valve (commonly used in medical fluid control) and the servo are calibrated to three discrete states: INLET (pressurization), OUTLET (depressurization) and HOLD (maintains pressure). Each servo provides 26 N cm of torque and switches states in 0.14 s per 60° rotation, enabling rapid transitions and precise control. The compact flow path minimizes dead volume and pressure losses, resulting in efficient and repeatable actuator performance with faster grasp–release cycles. Key advantages of the servo-driven valves are:

- Compact and lightweight: combines pressurization and depressurization in a single valve, cutting hardware footprint and weight, which is essential for wearable systems where comfort and portability are critical.
- Cost-effective: relies on inexpensive, widely available medical three-way valves and a single servo per channel, making it significantly cheaper than using high-speed industrial solenoid valves, especially when scaling to multiple actuators.
- Energy-efficient: the HOLD position maintains actuator pressure without continuously consuming compressed air or electrical power, reducing energy consumption and extending battery life.

Each valve channel is monitored by an integrated pressure sensor connected to an Arduino Mega microcontroller running the real-time control firmware. Grasp-intention signals from the EMG–inertial measurement unit (IMU) interface are transmitted wirelessly, enabling untethered operation. For patient safety, the control box also includes an emergency stop button and a manual depressurization valve that vents all actuators within seconds. Despite housing the compressor, valve manifold and electronics, the entire control box weighs about 2 kg and remains portable, allowing mounting on a wheelchair.

The finger flexor actuator was fabricated using a thermoplastic polyurethane bladder (radius 9 mm, tube length 620 mm) enclosed within a textile housing. The bladder was heat-sealed along its longitudinal edges and one extremity, with a pneumatic inlet integrated at the other end. The tube was subsequently inserted inside a textile nylon tube to prevent wall expansion. The resulting inflatable textile tube was enfolded into a fabric housing composed of 28 parallel stitched channels with a total length of 120 mm and channel width of 15 mm, thereby accommodating the folded segments and constraining radial expansion during pressurization. The wrist flexion and thumb abduction/opposition actuators were manufactured following the same procedure as the finger flexor actuator. Each actuator consists of a thermoplastic polyurethane bladder (radius 9 mm, tube length 800 mm) inserted into a nylon textile sleeve to limit wall expansion. The inflatable tube was then enfolded into a fabric housing composed of 15 parallel stitched channels with a total length of 80 mm and channel width of 50 mm.

Supplementary Fig. 1 shows the evaluation of blocked-tip force for the two textile soft actuators used in the hand exoskeleton, highlighting the relationship between air pressure and the generated output force. Both actuators exhibit a hysteresis loop between pressurization and depressurization, reflecting energy losses from the viscoelasticity of the actuator material and internal friction of the textile layers. The wrist flexion, thumb opposition and abduction actuator generates nearly twice the blocked force of the finger-flexion actuator over the same pressure range. The finger flexion achieved a maximum blocked-tip force of approximately 19 N, which lies within the range of reported physiological finger flexion forces of healthy individuals during activities of daily living (1.4–34.8 N)^34^. This indicates that the device can deliver effective assistance while remaining within a safe range relative to natural human capabilities. By contrast, wrist flexion, thumb opposition, and abduction generated forces of up to 37 N, slightly exceeding the upper bound of reported finger forces during activities of daily living. Such elevated force output may be advantageous for high-assist scenarios and supports moving the larger biomechanical structures around the wrist and carpometacarpal joint of the thumb.

### Silicon hand model

We found that evaluating hand exoskeleton designs for assisting almost completely paralysed individuals is impossible with healthy persons as they will always contribute forces during grasping, even when instructed to keep their hands passive. Thus, grasp motions that work well with healthy participants often do not work with severely impaired or paralysed patients. As a solution, we build a dummy hand by replacing the rigid metal threads between the bones of an educational hand model with elastic cords, thus allowing the fingers to move passively. The skeletal hand was then inserted inside a rubber glove made of latex, which was later filled with silicon (Ecoflex 00-30) to replicate the shape and size of a human hand. The weight of the silicon hand model is 400 g and thus closely matches the weight of an average human hand (0.585% of body mass^35^ and thus 440 g for a person of 75 kg). For our grasp robustness experiments (Fig. 1b), we placed this silicon hand model inside the hand exoskeleton and attached it to a mannequin arm.

### Myoelectric control interface for intentional grasping

To enable the patient to control the exoskeleton grasps, we first tested a wireless button that he could press with his second hand and a wireless foot pedal. While both allowed robust control, they had the drawback of being unintuitive to the patient as they involved muscles that he would not normally use for grasping and introduced a control delay due to the time of the hand/foot movement required to press them. The patient’s preferred intentional control method was an sEMG-based approach where he could use residual muscle activity in his flexor pollicis longus to initiate grasps, hold a desired grasp force and initiate hand opening. The sEMG signals were recorded following the SENIAM (Surface ElectroMyoGraphy for the Non-Invasive Assessment of Muscles) recommendations ^36^ with a Delsys Trigno Quattro wireless sensor (DelsysAPI 2.1 with Python 3.8) placed over the flexor pollicis longus muscle on his right hand after preparing the skin with an alcohol swab to remove surface oils and other contaminants. The reference electrode was placed on the proximal part of the brachioradialis where no sEMG signal could be detected and the patient could not perform associated pronation or supination. The analogue differential signal was measured in the range of ±5 V, digitized at a sampling rate of 1,111 Hz, and bandpass-filtered between 20 and 450 Hz to capture the main frequency range of physiological muscle activity and remove d.c. offset and high-frequency noise. Single-value spikes in the signal were replaced by interpolated signal values. We then computed the RMS signal over sliding windows of 250 ms. To detect intentional EMG activity onset, we make use of an adaptive thresholding algorithm^37,38^. To detect whether a muscle activity onset is occurring at a given point in time, we first define the last 180 samples (162 ms time window) as the ‘activity window’ and the 1,111 samples (1 s time window) directly before the ‘reference window’. With a frequency of 37 Hz given by the hardware application programming interface, the mean of the bandpass-filtered RMS signal of the activity window is computed and compared with the mean and s.d. of the reference window directly before. The length of the activity window was chosen based on the patient’s prolonged EMG activity duration in our calibration recording. If the mean in the activity window is significantly higher than the mean in the reference window (based on the reference window signal’s s.d.), an EMG activity onset and, thus, an action intention is predicted (Fig. 4b).

1 $${y}_{\mathrm{grasp}}=\left\{\begin{array}{l}1\,\mathrm{for}\,\overline{{x}_{\mathrm{act}}} > \overline{{x}_{\mathrm{ref}}}+\tau{\sigma }_{\mathrm{ref}}\\ 0\,\mathrm{for}\,\overline{{x}_{\mathrm{act}}}\le \overline{{x}_{\mathrm{ref}}}+\tau{\sigma }_{\mathrm{ref}}\end{array}\right.{.}$$

Thus, at every point in time, the actual threshold value is based on the statistics of the moving reference time window, making it robust to temporarily increased noise and signal non-stationarities, for example, due to muscle fatigue or changing skin conductance. The inability of the patient to produce a strong EMG activity due to ALS, combined with movement-related artefacts during reaching movements in pick-and-place actions with the hand exoskeleton, resulted in a low SNR. To improve the system’s performance, we added contextual motion information about the action’s current state. The specific type of pick-and-place action was split into the sequential states of rest, reaching, grasping, holding, placing and releasing, which were tracked by the system with motion sensors (IMUs) and used to adapt the interface behaviour (Fig. 4c). During the reaching and placing states, our system inhibits the prediction of intentional grasping to avoid false positives due to motion artefacts, possibly leading the patient to drop an object. In the rest state, the patient could start the exoskeleton grasping by exceeding the adaptive threshold with his muscle activation. To keep the sessions short and avoid lengthy parameter searches, we prebuilt a prediction model with parameters selected for a healthy participant as a baseline model. The activity and reference window lengths were kept unchanged for the patient, but due to weak and noisy muscle signals, we increased the model’s sensitivity (Fig. 6b).

### ML-based error monitoring and correction

Selecting the adaptive threshold parameter to achieve a high sensitivity resulted in multiple false positives in the rest phases due to the patient’s small SNR and associated high baseline signal variability. Such false positives would have led to erroneous exoskeleton grasps during times when the patient did not intend to grasp. To detect and correct these errors, we trained a 1D CNN architecture for classification of rest phase versus muscle activity in 200-ms temporal windows with a stride of 1 on the first two EMG-RMS datasets from our patient with ALS. These windows were labelled ‘grasp-intention’ when they were between −200 ms and +800 ms around EMG-RMS signal peaks, while all other windows were labelled ‘rest’. For training, the data were randomly split at the window level (80% training, 20% validation) and stratified to preserve class distribution. The model architecture consisted of three sequential 1D convolutional layers (32, 64 and 128 filters, kernel size 3) with batch normalization and ReLU activation, followed by global average pooling and three fully connected layers (of 128, 64 and 32 neurons, respectively, projecting to a single binary output) with ReLU activation and dropout regularization (0.3) to prevent overfitting. For the training process, we utilized a weighted binary cross-entropy loss function with class balancing to address the inherent class imbalance between grasp-intention and rest samples, an Adam optimizer with a learning rate of 0.001, gradient clipping (maximum norm 1.0) and early stopping based on validation performance. In our error monitoring and correction approach, we applied this trained classification model to the test EMG-RMS dataset in parallel to the activity onset detection. The test dataset was a held-out session that was recorded after the training data recording, thus mimicking the anticipated real-world application of fitting a model to a patient’s individual signal characteristics and then employing it afterwards. In addition, this training–test split helped to avoid data leakage. If the majority of samples in the time interval of 200–500 ms after a detected onset were classified as ‘rest’, we labelled the preceding onset detection as a false positive, enabling us to stop the exoskeleton grasping responsively.

### Reporting summary

Further information on research design is available in the Nature Portfolio Reporting Summary linked to this article.

## Supplementary information

Supplementary Information Supplementary Fig. 1 and Tables 1 and 2.

Reporting Summary

Peer Review File

Supplementary Video 1 Using a silicone hand model replicating the passive mechanics of the patient’s paralysed hand, we performed extended repetitive grasp-and-lift experiments to evaluate the robustness and reliability of the soft hand exoskeleton without fatiguing the patient. The video demonstrates repeated execution of pinch, cylindrical, spherical and lateral grasps over hundreds of cycles, highlighting stable grasp performance across diverse object shapes and grasp configurations.

Supplementary Video 2 To enable intuitive intentional control of the soft hand exoskeleton, we developed EMG-based training games in Unity3D that allowed the patient to learn how to reliably trigger grasp commands using residual muscle activity. The video demonstrates the real-time EMG processing pipeline, including raw EMG acquisition, RMS signal extraction, movement intention detection and interactive game-based feedback used to train and evaluate voluntary control performance.

Supplementary Video 3 This video demonstrates the importance of co-activating the thumb flexion and extension actuators to increase thumb stiffness during precise pinch grasping with the soft hand exoskeleton. Using the silicone hand model, the video compares thumb–index pinching of a small object with and without thumb extensor co-activation, showing that co-activation stabilizes the thumb posture and enables successful grasping.

Supplementary Video 4 This overview video introduces the clinical motivation and translational development of the soft robotic hand exoskeleton for a patient with severe hand paralysis due to ALS. It presents the patient’s initial hand impairment, the residual thumb muscle activity used for EMG-based intentional control, and the functional outcome achieved with the exoskeleton, including regained ability to grasp everyday objects and feed himself independently.

Supplementary Video 5 This video demonstrates the capability of the soft hand exoskeleton to perform diverse functional grasp types, including index–thumb pinch, cylindrical grasping and lateral grasping, using a silicone hand model and weighted everyday objects. The actuator-state plots illustrate the coordinated activation sequences of the actuators to achieve stable grasps across different object shapes and weights.

Supplementary Video 6 This video demonstrates repeated power-grasp experiments with the soft hand exoskeleton using a silicone hand model and cubic objects of different sizes and weights. The experiments highlight the repeatability and robustness of coordinated multi-actuator grasping during repeated grasp-and-release cycles, while the actuator-state plots illustrate the temporal activation sequence underlying stable power grasp formation.

Supplementary Video 7 This video demonstrates thumb–index pinching of a cylindrical water bottle using the soft hand exoskeleton and a silicone hand model. The experiments show that successful grasp stabilization requires coordinated co-activation of the thumb abduction and thumb opposition actuators, while the accompanying actuator-state plots illustrate the temporal control sequence enabling reliable repeated grasp-and-hold performance.

Supplementary Video 8 This video demonstrates that the soft hand exoskeleton can execute multiple grasp strategies for activities of daily living, including pinch, cylindrical, spherical and lateral grasps. The patient successfully manipulates diverse household objects, highlighting the versatility and functional adaptability of the exoskeleton for assistive grasping tasks.

Supplementary Video 9 This video presents the BBT as a functional evaluation of repetitive pick-and-place movements using the soft hand exoskeleton. The patient was able to transfer up to five blocks within 60 s, demonstrating restored grasping and object manipulation capabilities.

Supplementary Video 10 This video demonstrates active wrist dorsiflexion supporting the soft hand exoskeleton during functional grasping tasks with heavy and large objects. The integrated wrist assistance enables stable holding of a 400-g cube and a 1-kg wooden stick, highlighting the importance of coordinated wrist and hand actuation for robust object manipulation.

Supplementary Video 11 This video presents the functional validation of the soft hand exoskeleton using selected ARAT tasks performed by patients with stroke with different impairment levels. The demonstrations include grasping and transferring a 10-cm cube, manipulating a large tube and performing precision pinch actions. Results show that both severely and moderately impaired patients could accomplish several gross grasp tasks, while precision manipulation performance varied depending on impairment severity. These experiments highlight the capability of the exoskeleton to support clinically relevant activities of daily living and standardized upper-limb assessment tasks.

Supplementary Video 12 This video demonstrates repeated two-finger pinching actions performed by the soft hand exoskeleton using different thumb–finger combinations, including thumb–little, thumb–ring, thumb–middle and thumb–index grasps. The actuator-state plot illustrates the coordinated timing of thumb flexion, thumb opposition and individual finger flexor activations required to achieve each pinch configuration. These experiments demonstrate the dexterity and reconfigurability of the exoskeleton for multifinger precision manipulation tasks.

Supplementary Video 13 This experiment evaluates the repeatability of preshaping and grasp execution of the soft hand exoskeleton across multiple ARAT manipulation tasks. Each task was repeated ten times to assess consistency, robustness and execution speed for different object geometries and grasp strategies. The reported average completion times indicate stable and repeatable task execution across trials, demonstrating reliable preshaping, grasp adaptation and object manipulation capabilities of the exoskeleton.

## Data Availability

Data related to this article are available via Zenodo at 10.5281/zenodo.20037933 (ref. ^39^).

## Code availability

Code for model training and analysis is available via Zenodo at 10.5281/zenodo.20037933 (ref. ^39^).
