# Supplementary material for: A dexterous soft hand exoskeleton restores intentional grasping in individuals with severe hand impairment
Source: Nat Mach Intell. 2026 Jun 23;8(7):1100–14. doi: 10.1038/s42256-026-01263-3 (PMC13395625; doi:10.1038/s42256-026-01263-3)
Supplement: Supplementary file 1 — Supplementary Fig. 1 and Tables 1 and 2. [file 42256_2026_1263_MOESM1_ESM.pdf]

nature machine intelligence

Supplementary information

https://doi.org/10.1038/s42256-026-01263-3

A dexterous soft hand exoskeleton restores intentional grasping in individuals with severe hand impairment

In the format provided by the authors and unedited

Table 1 provides a comparative overview of recent soft robotic glove studies, highlighting differences in actuation type, control interface, and target user population.

Table 2 provides an overview of all participants.

Figure 1 shows the evaluation of blocked-tip force for the two textile soft actuators used in the hand exoskeleton, highlighting the relationship between air pressure and the generated output force.

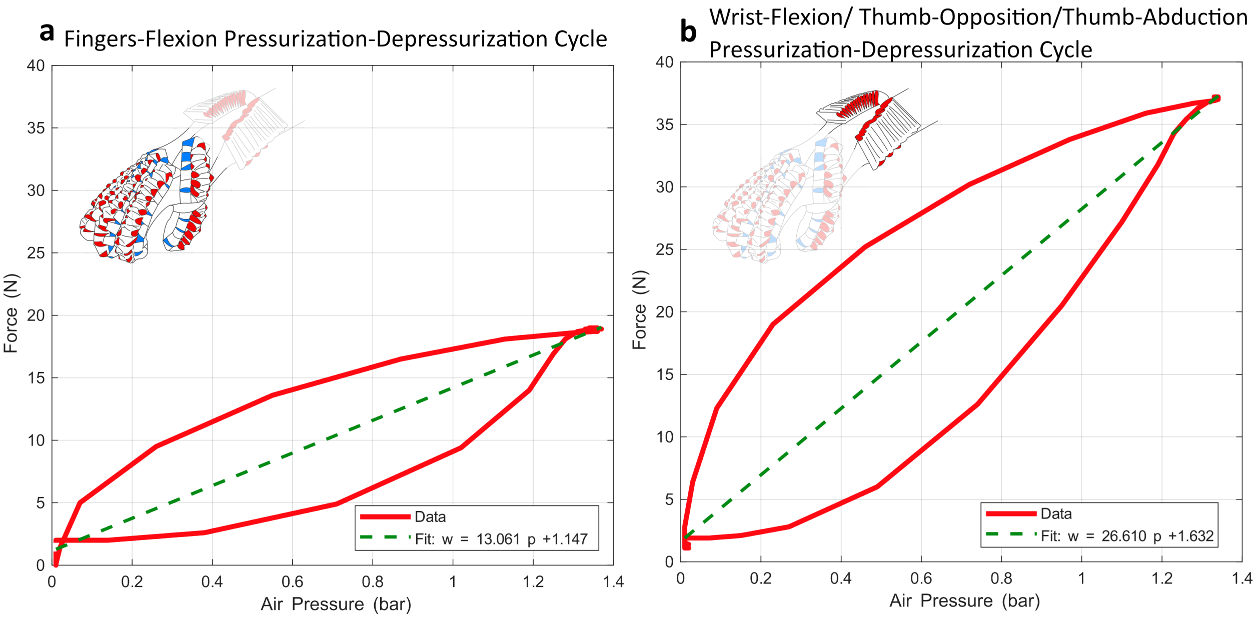

Figure 1: **Blocked-tip pressurization–depressurization cycles measured at 1 Hz for two types of textile soft actuators used in the hand exoskeleton.** **(a,)** The relationship between air pressure (bar) and generated tip force (N) during a full cycle with finger-flexion actuator. **(b,)** Due to their larger size, the wrist-flexion/ thumb-opposition/ thumb-abduction actuators can produce higher forces to move the larger biomechanical joint structures in the wrist and the thumb carpometacarpal joint. In both cases, the red curve represents the experimental pressurization–depressurization data, while the dashed green line shows the best linear fit.

Table 1: **Comparison of Soft Robotic Glove Studies.** Overview of actuation type, target population, clinical evaluation, control modality, and performance metrics. Ratings are assigned on a 1–5 scale, according to the following rubric: **Dexterity (1–5):** 1 = Single DoF (e.g., flexion only); 2 = Flexion + extension; 3 = Adds thumb motion (e.g., opposition or abduction); 4 = Adds wrist motion or multiple thumb DoFs; 5 = Full articulation: fingers, wrist, thumb (opposition + abduction), and co-activation. **Evaluation Scope (1–5):** 1 = No user tests; 2 = Limited lab test or small pilot; 3 = Basic task performance tests or few participants; 4 = Clinical scales (e.g., BBT, ARAT), functional evaluation with patients; 5 = Multi-method evaluation (clinical, functional, usability) with diverse users. **Severity Targeted (1–5):** 1 = Healthy users only; 2 = Mild impairment; 3 = Moderate; 4 = Severe (e.g., SCI, chronic stroke); 5 = Complete or near-complete paralysis (e.g., ALS, individuals with BBT score = 0).

Study	Actuation Type	Target Users	Clinical Tests	Control Interface	Dexterity (1–5)	Evaluation (1–5)	Severity (1–5)
Kang et al. (2019)	Tendon-driven	General assistive	Yes	Button/manual	2	3	2
Kottink et al. (2022)	Unspecified (soft)	Grip impairment	Protocol only	Not specified	1	2	2
Ge et al. (2020)	Pneumatic (fabric)	Brachial plexus injury	Lab testing	Manual	3	3	2
Lim et al. (2023)	Pneumatic (bidirectional)	Stroke (chronic)	Pilot trials	Manual	2	4	4
Cappello et al. (2018)	Tendon-driven (fabric)	SCI (C4–C7)	ADL trials	Manual	2	4	4
Kim et al. (2022)	Cable-driven	General assistive	Bench + user test	Manual	4	3	3
Chen et al. (2022)	Motorized orthosis	Stroke (hemiparesis)	Task evaluation	Button	3	4	3
This work	Pneumatic textile-based	ALS + Stroke (BBT=0)	BBT, ARAT, SUS	EMG + IMU	5	5	5

Table 2: **Study participants** Our primary participant P1, who participated in the co-creation process, had a severe hand impairment due to ALS. To validate our soft hand exoskeleton with a larger group of patients, we recruited six individuals with hemiparetic hand impairment due to an ischemic middle cerebral artery stroke in our study. Three of the stroke patients were equally severely impaired as our co-creation patient P1 (Box-and-Blocks Test score 0), whereas the other three stroke patients had moderate hand impairments.

Participant	Neurological Condition	Age	Gender	Side of Paresis	Chronicity	BBT Score
P1	ALS	65	male	both	4 years	0
P2	Stroke	80	male	left	16 weeks	0
P3	Stroke	83	female	right	18 weeks	19
P4	Stroke	80	female	left	4 weeks	6
P5	Stroke	81	female	right	15 weeks	0
P6	Stroke	61	male	right	5 weeks	0
P7	Stroke	79	female	right	8 weeks	12
