# Supplementary material for: A dexterous soft hand exoskeleton restores intentional grasping in individuals with severe hand impairment
Source: Nat Mach Intell. 2026 Jun 23;8(7):1100–14. doi: 10.1038/s42256-026-01263-3 (PMC13395625; doi:10.1038/s42256-026-01263-3)
Supplement: Supplementary file 2 — Reporting Summary [file 42256_2026_1263_MOESM2_ESM.pdf]

nature portfolio

Corresponding author(s): Gordon Cheng

Last updated by author(s): May 13, 2025

Reporting Summary

Nature Portfolio wishes to improve the reproducibility of the work that we publish. This form provides structure for consistency and transparency in reporting. For further information on Nature Portfolio policies, see our Editorial Policies and the Editorial Policy Checklist.

Statistics

For all statistical analyses, confirm that the following items are present in the figure legend, table legend, main text, or Methods section.

n/a	Confirmed	
☐	☒	The exact sample size (*n*) for each experimental group/condition, given as a discrete number and unit of measurement
☐	☒	A statement on whether measurements were taken from distinct samples or whether the same sample was measured repeatedly
☐	☒	The statistical test(s) used AND whether they are one- or two-sided<br>*Only common tests should be described solely by name; describe more complex techniques in the Methods section.*
☒	☐	A description of all covariates tested
☐	☒	A description of any assumptions or corrections, such as tests of normality and adjustment for multiple comparisons
☐	☒	A full description of the statistical parameters including central tendency (e.g. means) or other basic estimates (e.g. regression coefficient) AND variation (e.g. standard deviation) or associated estimates of uncertainty (e.g. confidence intervals)
☐	☒	For null hypothesis testing, the test statistic (e.g. *F*, *t*, *r*) with confidence intervals, effect sizes, degrees of freedom and *P* value noted<br>*Give P values as exact values whenever suitable.*
☒	☐	For Bayesian analysis, information on the choice of priors and Markov chain Monte Carlo settings
☒	☐	For hierarchical and complex designs, identification of the appropriate level for tests and full reporting of outcomes
☒	☐	Estimates of effect sizes (e.g. Cohen's *d*, Pearson's *r*), indicating how they were calculated

Our web collection on statistics for biologists contains articles on many of the points above.

Software and code

Policy information about availability of computer code

Data collection	The sEMG signals were recorded with a Delsys Trigno Quattro wireless sensor using the DelsysAPI interface in Python (https://github.com/delsys-inc/Example-Applications). (DelsysAPI 2.1 with Python 3.8)
Data analysis	Data analysis was performed with Python version 3.12.1 using the following libraries: pandas 2.2.3, numpy 1.26.4, matplotlib 3.10.6, joblib 1.5.1, torch 2.7.1, scikit-learn 1.7.0, scipy 1.16.0. Custom code for the data analysis can be found at https://github.com/TUM-ICS/EMG-soft-hand-exoskeleton

For manuscripts utilizing custom algorithms or software that are central to the research but not yet described in published literature, software must be made available to editors and reviewers. We strongly encourage code deposition in a community repository (e.g. GitHub). See the Nature Portfolio guidelines for submitting code & software for further information.

Data

Policy information about availability of data

All manuscripts must include a data availability statement. This statement should provide the following information, where applicable:

- Accession codes, unique identifiers, or web links for publicly available datasets
- A description of any restrictions on data availability
- For clinical datasets or third party data, please ensure that the statement adheres to our policy

Data is available at https://github.com/TUM-ICS/EMG-soft-hand-exoskeleton

Research involving human participants, their data, or biological material

Policy information about studies with human participants or human data. See also policy information about sex, gender (identity/presentation), and sexual orientation and race, ethnicity and racism.

Reporting on sex and gender	In Table 2 we report the self-assigned gender of all study participants. While effects of sex and gender were not central to our study design, we paid attention to not include patients from exclusively one sex or gender.
Reporting on race, ethnicity, or other socially relevant groupings	The study design did not consider race, ethnicity, or other socially relevant groupings. As reported in Table 2, all participants were aged 60 years or older. This age distribution emerged naturally during recruitment consistent with the well-established age-related incidence of stroke and ALS and was not an explicit selection criterion.
Population characteristics	All participants had neurological conditions affecting hand function. The primary co-creation participant was a 65-year-old individual diagnosed with amyotrophic lateral sclerosis (ALS) with severe distal tetraparesis and minimal residual thumb movement, representing a high-impairment profile. The six stroke participants were adults aged 61-83 years who had experienced an ischemic middle cerebral artery stroke several weeks prior to recruitment and were undergoing early neurorehabilitation. Their hand impairments ranged from severe to moderate. None of the participants had cognitive, sensory, or communication deficits that would limit study participation. No additional covariate-relevant characteristics (e.g., genotypic information or past medical history beyond stroke/ALS diagnosis) were collected, as they were not required to address the study aims.
Recruitment	The ALS patient was recruited at a rehabilitation hospital based on his tetraparesis with almost complete hand paralysis while having a relatively stable disease progression that would allow our co-creation study over multiple months. The stroke patients were recruited in a rehabilitation hospital based on moderate or severe hand impairment without cognitive or communication deficits.<br>Self-selection bias is unlikely to have meaningfully influenced the results, as participants were consecutively enrolled from clinical settings and inclusion was not dependent on patient initiative. However, there might be a potential bias in patient motivation to have an improved functional performance between the two stroke patient groups.
Ethics oversight	The study was approved by the Ethics Board of the Technical University of Munich Hospital, Klinikum Rechts der Isar<br>Reference number: 167/21 S-EB

Note that full information on the approval of the study protocol must also be provided in the manuscript.

Field-specific reporting

Please select the one below that is the best fit for your research. If you are not sure, read the appropriate sections before making your selection.

☒ Life sciences ☐ Behavioural & social sciences ☐ Ecological, evolutionary & environmental sciences

For a reference copy of the document with all sections, see nature.com/documents/nr-reporting-summary-flat.pdf

Life sciences study design

All studies must disclose on these points even when the disclosure is negative.

Sample size	No sample-size calculation was performed, as this was a translational proof-of-concept study and participant numbers were determined by the availability of eligible ALS and stroke patients.
Data exclusions	no data was excluded from analysis
Replication	To validate the hand exoskeleton's assistive benefits during grasping after finishing the co-creation phase with the ALS patient, we included six additional stroke patients for replication across subjects. The successful replication of the soft hand exoskeleton's usage was demonstrated in all six stroke patients. As reported in Figure 5, the assistive effect of the exoskeleton was dependent on the severity of the impairment and the specific task.
Randomization	The stroke patients were allocated to the moderately or severely impaired groups based on their box-and-blocks test performance. Patients who could not move a single block were allocated to the severely impaired group. To control for training or fatigue effects, we randomized the sequence of assisted and non-assisted grasping trials.
Blinding	During the validation experiments with stroke patients, the investigators were not blinded to the patients allocation to the moderately and severe hand impairment groups. Stroke patients who scored zero in the ARAT grasp, grip and pinch subsets were allocated to the 'strongly impaired' group. Investigators could not be blinded to their allocation due to their clinically apparent functional and behavioral limitations. However, blinding was not relevant to the study outcomes as the primary measure was objective functional assessment when assisted by the exoskeleton.

Reporting for specific materials, systems and methods

We require information from authors about some types of materials, experimental systems and methods used in many studies. Here, indicate whether each material, system or method listed is relevant to your study. If you are not sure if a list item applies to your research, read the appropriate section before selecting a response.

Materials & experimental systems

n/a	Involved in the study
☒	☐ Antibodies
☒	☐ Eukaryotic cell lines
☒	☐ Palaeontology and archaeology
☒	☐ Animals and other organisms
☒	☐ Clinical data
☒	☐ Dual use research of concern
☒	☐ Plants

Methods

n/a	Involved in the study
☒	☐ ChIP-seq
☒	☐ Flow cytometry
☒	☐ MRI-based neuroimaging

Plants

Seed stocks	n/a
Novel plant genotypes	n/a
Authentication	n/a
