# Supplementary material for: A dexterous soft hand exoskeleton restores intentional grasping in individuals with severe hand impairment
Source: Nat Mach Intell. 2026 Jun 23;8(7):1100–14. doi: 10.1038/s42256-026-01263-3 (PMC13395625; doi:10.1038/s42256-026-01263-3)
Supplement: Supplementary file 3 — Peer Review File [file 42256_2026_1263_MOESM3_ESM.pdf]

nature portfolio

Peer Review File

A Dexterous Soft Hand Exoskeleton Restores Intentional Grasping for Individuals with Severe Hand Impairment

Corresponding Author: Professor Gordon Cheng

This file contains all reviewer reports in order by version, followed by all author rebuttals in order by version.

Version 0:

Reviewer comments:

Reviewer #1

(Remarks to the Author)
This paper presents a carefully executed example of translational soft robotics applied to severe hand impairment. The co-creation approach with a patient affected by advanced ALS is a major asset, as it grounds the engineering choices in real clinical needs and leads to a device that prioritizes comfort, safety, perceived control, and meaningful functional outcomes. The resulting soft hand exoskeleton, with its multi-articulate design including an opposable and abductable thumb and wrist dorsiflexion, clearly goes beyond simple strength augmentation and tackles dexterity and preshaping, which are critical for everyday grasping. The demonstration that the primary participant regained the ability to intentionally grasp diverse objects and feed himself is compelling and highlights the potential real-world impact of the work

The integration of an sEMG-based intentional control interface, combined with motion context and error monitoring, is another strong contribution. The authors convincingly show that even extremely weak and noisy EMG signals can be exploited to provide a sense of agency, which is often as important as raw functional gain in assistive devices. The comparison with healthy controls and the personalization of the control strategy strengthen the technical credibility of the approach. The additional validation in stroke patients, particularly the differential benefit observed in severely versus moderately impaired individuals, provides useful insight into which patient groups are most likely to benefit from such a high-dexterity solution.

Please address the following open issues:

1. Please clarify more explicitly the intended clinical target population, distinguishing between patients with near-complete paralysis and those with moderate residual function, and reflect this distinction consistently in the abstract, discussion, and conclusions.
2. Consider adding a short paragraph that contextualizes the single-patient co-creation study within a translational framework, explicitly stating what aspects are exploratory, what are demonstrative, and which findings should not yet be generalized.
3. Expand the discussion on limitations related to scalability and deployment, including system complexity, pneumatic actuation, calibration needs, and feasibility of routine clinical or home use, to better frame the remaining translational gap.
4. Please clarify the clinical meaning of the reported outcome measures (BBT, ARAT changes), especially for severely impaired patients, by explicitly linking score changes to functional relevance in activities of daily living.
5. The results obtained in moderately impaired stroke patients warrant a more explicit interpretation; please discuss whether this reflects a mismatch between device design and impairment level, task constraints, or control strategy limitations.
6. Consider adding a brief comparison with existing clinically available assistive devices, even if less dexterous, to better position the proposed system relative to current standards of care.
7. A short forward-looking paragraph outlining the next necessary steps toward clinical translation would be valuable, for example larger controlled trials, simplification of hardware, regulatory considerations, or adaptation to progressive disease trajectories.

Minor editorial suggestion: some sections of the discussion partially reiterate results; these could be streamlined to sharpen the take-home message and emphasize translational insight rather than technical detail.

Reviewer #2

(Remarks to the Author)
In this work, Nassour and colleagues present a novel dexterous soft hand exoskeleton developed with direct input from a patient with severe upper-limb impairment due to ALS. The device was evaluated in this individual as well as in six additional participants with moderate-to-severe hand paralysis following stroke.

Overall, the exoskeleton appears to improve hand dexterity and functional performance during activities of daily living. Importantly, the integration of end-user feedback throughout the design process is a major strength and is executed in a thoughtful and rigorous manner. However, I have three main concerns.

First of all, the exoskeleton was tested in patients with different neurological disorders: ALS, SMA, stroke. However, in the design only feedback from the ALS patient were taken into consideration. Hand paralysis due to different neurological causes results in different symptoms. For instance, stroke patients often have a spastic hand close in a fist. This is quite different from hand weakness in ALS and SMA patients. This difference might require adjustment in the design of the exoskeleton that were not taken into consideration in this work. Moreover, it is unclear why the SMA participant only performed the EMG tests and they didn't test the exoskeleton.

Second, the author commented that to be effective in daily living an exoskeleton "should be soft and lightweight, intuitively controlled, and capable of supporting the grasp of objects with varying shapes and weights". Several of these characteristics have not been demonstrated true in the current design. For instance, the description of the weight should be reported in the main results and compare to other similar devices. The "intuitively controlled" should be demonstrated comparing EMG control versus button trigger control in terms of motor performances over different participants and sessions. Testing of the exoskeleton control paired with some cognitive tasks is also often used to demonstrate the intuitive control of a device. Finally, no tests with objects with varying weights were performed.

Finally, while the author did a very good job on showing how the exoskeleton can improve performances in the BBT task, the manuscript lacks data on supporting reliability and robustness of the results. Here a few examples where more data would be necessary to support the authors conclusions. For instance, in the tests with the 2 intact participants, the authors reported that all grasp types resulted in more than 125 successful grasp-and-lift actions. However, it is unclear how many times did the grasps fail. Moreover, it would be interesting to see the reliability of repetitions of the pre-shaping and grasping. Can the exoskeleton detect and replicate these positions over several repetitions? It would also be important to see how positioning of the hand (for instance movement against vs. towards gravity) affects performances. The same analysis (maybe with a few less grasp/hand gesture because of fatigue) should be repeated for the patients. Indeed, patients' performances are often very variable, and this can affect robot performances. Moreover, it would be important to understand whether the EMG control would need to be recalibrate over sessions. This is usually a big drawback of EMG/neural control systems. The authors also reported that the "the co-created design changes enabled the ALS participant to reliably grasp a bottle (cylindrical grasp), a plate (lateral grasp), an apple (spherical grasp), and a pastry (pinch grasp), thus demonstrating the capability of the hand exoskeleton to support multiple grasp types for diverse objects of everyday life. None of these data were reported in the manuscript. Finally, it is unclear why stroke participants have not been tested in similar daily living tasks.

Minor comment: The organization of the Introduction is somewhat unusual. It begins by presenting the problem, then introduces the authors' solution, and subsequently returns to the problem statement. This structure makes the narrative difficult to follow. In addition, including figures in the Introduction is uncommon. I suggest revising this section to improve linearity and clarity.

Version 1:

Reviewer comments:

Reviewer #1

(Remarks to the Author)
The paper reports the co-design and evaluation of a multi-articulate, pneumatically actuated soft hand exoskeleton targeting individuals with severe to near-complete hand paralysis, primarily an ALS patient with a pre-intervention BBT score of zero. The central technical contribution is the integration of an active opposable and abductable thumb with wrist dorsiflexion support, filling a well-documented gap in the soft exoskeleton literature, where most devices are limited to simple flexion-extension and target moderately impaired users.

The paper has been significantly improved. Here some additional issues:

1. As pointed out, the entire design process rested on a single ALS patient, and that stroke-related spasticity presents very different mechanical and control requirements. The authors' response is honest: they reframe the co-creation study as feasibility-demonstrative rather than population-representative, explicitly carve out stroke patients and the SMA participant as validation rather than co-design contributors, and add appropriate caveats in the abstract, introduction, and discussion. This is the right framing, though it also makes the claim of "broader applicability" correspondingly more modest. The authors

should work on this.

2. Robustness and reliability of the data was an issue we raised before. The authors add a grasp success/failure table (overall accuracy 95–98% across grasp types in healthy controls), a repeatability supplementary video including a water-pouring task, and cross-session EMG generalization analysis. These additions meaningfully strengthen the paper. However, no patient-level repeatability data is provided across sessions, which remains an important open question, particularly given the progressive nature of ALS and the acknowledged need for periodic threshold recalibration.

3. Claims about intuitiveness and weight: the authors appropriately remove the term "intuitively controlled" and replace it with "hands-free and intention-driven," and acknowledge the absence of cognitive dual-task validation. This is a reasonable scope correction. The weight issue (352 g glove + ~2 kg control box) is acknowledged as a deployment barrier, though the 2 kg compressor-valve-electronics unit is a non-trivial portability limitation that may deserve more emphasis given the intended home-use aspiration.

4. The most significant unresolved limitation is that the exoskeleton is, by construction, calibrated to one individual's anatomy, residual function, and disease stage. The nine-month co-creation process is a strength methodologically but also means that design choices — thumb geometry, actuator pressures, electrode placement — may not transfer without significant re-engineering to other ALS patients, even at comparable disease stages. The validation cohort (n=6 stroke patients, one ALS co-creator) is too small to draw population-level conclusions, which the authors now acknowledge explicitly.

(Remarks on code availability)

Reviewer #2

(Remarks to the Author)
I would like to thank the authors for adressing all my comments and modifying the manuscript accordingly. There is still one point that needs to be addressed. I appreciate the addition of the supplementary video to show repeatability of preshaping and grasp execution. However, I believe it would be important to quantify how replicable and successful were the different tasks.

(Remarks on code availability)

Open Access This Peer Review File is licensed under a Creative Commons Attribution 4.0 International License, which permits use, sharing, adaptation, distribution and reproduction in any medium or format, as long as you give appropriate credit to the original author(s) and the source, provide a link to the Creative Commons license, and indicate if changes were made.
In cases where reviewers are anonymous, credit should be given to 'Anonymous Referee' and the source.
The images or other third party material in this Peer Review File are included in the article’s Creative Commons license,

unless indicated otherwise in a credit line to the material. If material is not included in the article's Creative Commons license and your intended use is not permitted by statutory regulation or exceeds the permitted use, you will need to obtain permission directly from the copyright holder.
To view a copy of this license, visit https://creativecommons.org/licenses/by/4.0/

Response to the Reviewers

Manuscript Title:
A Dexterous Soft Hand Exoskeleton Restores Intentional Grasping for Individuals with Severe Hand Impairment

Journal: Nature Machine Intelligence

We thank the Editor and the Reviewers for their careful evaluation of our manuscript and for their constructive and insightful comments. We are pleased that both reviewers found the work to be of interest and recognized the translational relevance, co-creation methodology, and clinical potential of our approach. Below, we address each comment point by point. All changes are reflected in the revised manuscript with tracked changes enabled, and relevant section references are provided for clarity.

Response to Reviewer #1

Comment 1: *1. Please clarify more explicitly the intended clinical target population, distinguishing between patients with near-complete paralysis and those with moderate residual function, and reflect this distinction consistently in the abstract, discussion, and conclusions.*

Response: We thank the reviewer for this important suggestion. We have clarified the intended clinical target population and now explicitly distinguish between individuals with severe to near-complete hand paralysis and those with moderate residual function.
The exoskeleton was primarily designed through a co-creation process with an individual with near-complete paralysis due to ALS, and its dexterity, control strategy, and bio-inspired preshaping mechanisms specifically address the needs of patients who cannot preshape or stabilize their hand prior to grasping.
We have revised the abstract, discussion, and conclusions to consistently reflect that the system is principally intended for patients with severe impairments, while the cohort with moderate residual function serves to highlight impairment-level-dependent utility and limitations. These revisions also emphasize that assistive benefit and usability decrease when the exoskeleton is applied outside its intended severity range.

We added to the abstract: "These results indicate that the soft hand exoskeleton is particularly effective for individuals with severe to near-complete hand paralysis, while its utility for patients with moderate residual function is limited and task-dependent."

In the introduction, we replaced: "This validation with stroke patients confirmed that patients with severe impairments benefit the most, highlighting importance of adapting assistive exoskeleton designs to patient impairment levels."
BY:
"In this work, we explicitly target individuals with severe to near-complete hand paralysis, for whom preshaping and grasp stabilization cannot be achieved voluntarily, and we contrast their

outcomes with those of patients with moderate residual function to delineate the boundaries of assistive benefit."

In results, we added: "Importantly, this stratification was intentional, as the exoskeleton was not designed to augment fine motor control in patients with moderate residual function, but rather to restore basic grasping capability in those with severe paralysis."

In discussion, we added: "A key finding of this study is that the utility of assistive hand exoskeletons is strongly dependent on the level of residual hand function. While patients with severe impairments benefited substantially from the additional dexterity and preshaping support, some moderately impaired patients experienced no benefit or even reduced performance. This highlights that exoskeletons optimized for severe paralysis may interfere with voluntary motor strategies in patients with higher residual function, underscoring the need for impairment-specific assistive designs."

And in conclusion, we replaced: "In conclusion, by following a co-creation approach, we designed a multi-articulate soft hand exoskeleton with which a patient with severe hand function impairment showed significant functional improvement in grasp-and-lift actions, eventually enabling him to feed himself."
BY:
"In conclusion, we present a co-created, multi-articulate soft hand exoskeleton designed specifically for individuals with severe to near-complete hand paralysis, demonstrating that restoring dexterity, rather than merely augmenting strength, is critical for this adult patient population."

Comment 2: *2. Consider adding a short paragraph that contextualizes the single-patient co-creation study within a translational framework, explicitly stating what aspects are exploratory, what are demonstrative, and which findings should not yet be generalized.*

Response: We thank the reviewer for this suggestion. In response, we added a paragraph in the discussion that contextualizes the single-patient co-creation study within a translational framework, explicitly distinguishing exploratory design aspects from demonstrative outcomes and clearly stating the limits of generalization.

We added "Within a translational framework, the single-patient co-creation study should be interpreted as both exploratory and demonstrative. The iterative design process, bio-inspired actuation strategies, and intentional control interface were explored and refined exclusively in close collaboration with one individual with near-complete ALS-related hand paralysis, and therefore primarily demonstrate feasibility rather than population-level efficacy. At the same time, the resulting ability to reliably perform diverse grasp types and to regain a meaningful activity of daily living, namely independent feeding, serves as a demonstrative proof of principle for what multi-articulate soft hand exoskeletons can enable when tailored to severe impairment. Findings related to control performance, usability, and functional gains should not yet be generalized beyond this impairment level or design configuration, motivating the subsequent

validation with stroke patients and future studies with larger cohorts."

Comment 3: *3. Expand the discussion on limitations related to scalability and deployment, including system complexity, pneumatic actuation, calibration needs, and feasibility of routine clinical or home use, to better frame the remaining translational gap.*

Response: We thank the reviewer for highlighting this important point. We have expanded the Discussion to explicitly address limitations related to system complexity, pneumatic actuation, calibration requirements, and feasibility of routine clinical or home deployment, thereby clarifying the remaining translational gap and outlining directions for future development.

We added this paragraph to the discussion: "Several limitations related to scalability and deployment remain and frame the current translational gap of the presented system. First, the high level of dexterity required to assist individuals with severe to near-complete hand paralysis results in increased system complexity, including multiple pneumatic actuators, valves, sensors, and control components, which currently limits ease of setup and maintenance. Second, pneumatic actuation, while lightweight, compliant, and well suited for soft wearable devices, requires an integrated air supply and introduces constraints related to portability, noise, and long-term robustness that must be addressed for routine clinical or home use. Third, the intentional control interface requires individual calibration and periodic adjustment to accommodate day-to-day variability in residual muscle activity, particularly in progressive conditions such as ALS, which currently necessitates expert supervision. Together, these factors indicate that while the system demonstrates translational feasibility and clinical relevance, further engineering efforts are required to reduce hardware complexity, automate calibration, and improve robustness before deployment outside supervised clinical environments."

And we added this sentence to the conclusion: "Bridging the remaining translational gap will require reducing system complexity, improving portability, and automating calibration to enable routine clinical and home deployment."

Comment 4: *4. Please clarify the clinical meaning of the reported outcome measures (BBT, ARAT changes), especially for severely impaired patients, by explicitly linking score changes to functional relevance in activities of daily living.*

Response: We thank the reviewer for this important point. We have expanded the Discussion to clarify the clinical meaning of changes in BBT and ARAT scores, explicitly linking non-zero scores and observed improvements in severely impaired patients to functionally meaningful abilities in activities of daily living.

We added this paragraph to the discussion:"The clinical meaning of changes in standard outcome measures such as the Box-and-Blocks Test (BBT) and the Action Research Arm Test (ARAT) depends strongly on the baseline level of impairment. For individuals with severe to near-complete hand paralysis, these assessments exhibit pronounced floor effects, such that non-zero scores reflect the emergence of a qualitatively new functional capability rather than

incremental improvement. In this context, the ability to transfer any blocks in the BBT or to complete grasp and grip items in the ARAT corresponds to regaining intentional object manipulation, which directly translates to activities of daily living such as holding utensils, stabilizing objects, or feeding. Thus, although absolute score changes remain modest, they represent functionally meaningful transitions from inability to capability for severely impaired patients."

We also added a sentence to the results, subsection(Grasping intention can be detected from non-invasive EMG signals despite low signal-to-noise ratio): "For the co-creation participant, achieving a non-zero BBT score corresponded to the ability to intentionally grasp, transport, and release objects, enabling independent feeding."

Comment 5: *5. The results obtained in moderately impaired stroke patients warrant a more explicit interpretation; please discuss whether this reflects a mismatch between device design and impairment level, task constraints, or control strategy limitations.*

Response: We have expanded the Discussion to explicitly interpret the results observed in moderately impaired stroke patients, discussing the role of impairment–design mismatch, task constraints of the ARAT, and limitations of the control strategy, and clarifying that the observed effects primarily reflect the application of a device optimized for severe impairment to patients with higher residual function.

We added to discussion: "The limited or negative performance changes observed in moderately impaired stroke patients likely reflect a combination of factors rather than a failure of the assistive approach. Notably, the present design does not explicitly address spasticity or abnormal muscle tone, which are common after stroke and may require distinct mechanical compliance, control strategies, or therapeutic integration. First, the exoskeleton was explicitly designed to restore grasping capability in individuals who cannot preshape or stabilize the hand voluntarily, and therefore its level of assistance and coordinated actuation may interfere with intact or partially intact motor strategies in patients with moderate residual function. Second, several ARAT tasks, particularly in the Grip and Pinch subsets, require fine motor coordination and adaptive force modulation, which may be constrained by the exoskeleton's predefined grasp patterns and compliance characteristics. Third, the intentional control strategy was optimized for users with minimal residual activity and prioritizes robustness and responsiveness over fine-grained modulation, which may limit its suitability for patients who can already initiate and modulate grasp movements voluntarily. Together, these factors suggest that the observed results primarily reflect a mismatch between device design and impairment level, while also highlighting task- and control-specific limitations that should be addressed in future impairment-adaptive exoskeleton systems."

We also added a sentence to the result (in the subsection: Validation of hand function assistance and usability with stroke patients): "These findings suggest that the assistive strategy is most effective when voluntary preshaping and grasp stabilization are absent, and may be less suitable when residual motor control is preserved."

Comment 6: *6. Consider adding a brief comparison with existing clinically available assistive devices, even if less dexterous, to better position the proposed system relative to current standards of care.*

Response: We thank the reviewer for this suggestion. We have added a brief comparison in the Discussion that contextualizes the proposed system relative to clinically available assistive devices, clarifying how its increased dexterity addresses a functional gap in current standards of care for patients with severe hand paralysis.

We added to discussion: "In current clinical practice, assistive options for impaired hand function primarily include passive orthoses, static or dynamic splints, and simple active devices that provide uniform finger flexion or extension, often controlled via buttons or switches. While such systems are comparatively robust and easier to deploy, their limited dexterity and lack of coordinated thumb opposition or preshaping support restrict their effectiveness, particularly for individuals with severe to near-complete hand paralysis who cannot voluntarily configure the hand prior to grasping. In this context, the proposed system does not seek to replace existing standards of care, but rather to address a functional gap for patients who derive limited benefit from less dexterous devices, demonstrating how increased articulation and adaptive control can restore intentional object manipulation when simpler assistive strategies are insufficient."

Comment 7: *7. A short forward-looking paragraph outlining the next necessary steps toward clinical translation would be valuable, for example larger controlled trials, simplification of hardware, regulatory considerations, or adaptation to progressive disease trajectories.*

Response:

Comment 8: *Minor editorial suggestion: some sections of the discussion partially reiterate results; these could be streamlined to sharpen the take-home message and emphasize translational insight rather than technical detail.*

Response: We have streamlined portions of the Discussion to reduce repetition of results and sharpen the take-home message, with greater emphasis on interpretation and translational insight.

In the discussion, We replaced: "In this study, we showed that augmenting a soft hand exoskeleton with an opposable thumb and bio-inspired actuation principles such as hand preshaping and stiffness adaptation through co-activation enabled an individual with severe hand impairment to grasp various objects of daily living. While EMG-based control was the preferred intentional control interface, the low SNR of our severely impaired patient's EMG signal resulted in a high number of false-positive detections. Adding a machine learning-based error monitoring and correction process to a fast onset detection approach significantly reduced the false-positives and enabled fast error recovery. Our ALS patient preferred this "trigger fast and correct after" method as it combined high responsiveness with reliable grasp intention prediction."

BY:

"In this study, we demonstrate that restoring hand dexterity through multi-articulate soft hand exoskeleton and impairment-adapted control can enable meaningful object manipulation in individuals with severe hand impairment, even in the presence of weak and noisy myoelectric signals."
Motivation: The removed paragraph re-describes system components and control performance which are already detailed in Results.

We also removed the text in the discussion: "Our results show that patients with severe hand impairment similar to our co-creation participant benefited most from the exoskeleton assistance during hand function tasks. While the hand patients with moderate hand impairment only exhibited marginal score improvements due to the exoskeleton's grip strength assistance, the patient's with severe hand impairment also benefited from our exoskeletons hand dexterity assistance which resulted in large ARAT score improvements. The results in the ARAT pinch subtasks show that our exoskeleton can improve pinching with different fingers due to its opposable thumb, however pinching assistance for tiny objects still remains a challenge. Additionally we observed a reduction of hand function scores for some of the moderately impaired patients, suggesting that exoskeletons designed with and for patients with severe hand impairments might not generalize well to patients with smaller impairments. This impairment level-dependence of exoskeleton utility was also reflected in the usability evaluations we obtained during the validation experiments."
Motivation: The removed text repeats that severe patients improved more than moderate, restates ARAT improvement patterns, and reiterates usability differences already explained earlier.

We also replaced the text in the discussion: "Our adaptation of the hand exoskeleton to the impairment level of our co-creation participant was reflected in the results we obtained in a validation experiment with two groups of stroke patients, one group with moderate hand impairment and one group with severe hand impairment. The hand exoskeleton assistance resulted in significant functional improvements for the severely impaired patients, but, depending on the task, only small improvement and even reductions in hand function for the moderately impaired patients. In addition, the severely impaired patients reported a higher level of usability. These validation results suggest that functional assistance and usability of exoskeletons are dependent on the target patients' impairment levels."
BY:
"Together, the validation results reinforce that assistive benefit and usability are strongly dependent on impairment severity, underscoring the importance of matching exoskeleton design to residual motor function. "
Motivation: to eliminates redundancy as this message was already stated earlier.

Response to Reviewer #2

Comment 1: *First of all, the exoskeleton was tested in patients with different neurological disorders: ALS, SMA, stroke. However, in the design only feedback from the ALS patient were taken into consideration. Hand paralysis due to different neurological causes results in different symptoms. For instance, stroke patients often have a spastic hand close in a fist. This is quite different from hand weakness in ALS and SMA patients. This difference might require adjustment in the design of the exoskeleton that were not taken into consideration in this work. Moreover, it is unclear why the SMA participant only performed the EMG tests and they didn't test the exoskeleton.*

Response (general): We thank the reviewer for highlighting these critical critical aspects. To ensure clarity, we address the different aspects of this comment separately below, namely (i) the scope of the exoskeleton design process across neurological conditions, (ii) different design implications for flaccid and spastic hand impairments, and (iii) the rationale for including the SMA participant exclusively in the EMG analyses.

Response for (i): The co-creation process was intentionally centered on a single individual with severe ALS-related hand paralysis, as the primary goal of this study was to explore how a high-dexterity soft hand exoskeleton could be designed to restore intentional grasping in the context of near-complete voluntary hand function loss. We therefore do not claim that the co-created design captures the full spectrum of impairments arising from different neurological etiologies.

Rather than serving as co-creation participants, individuals with other neurological conditions were included at later stages of the study for targeted validation and comparison. Stroke patients with different levels of residual hand function were recruited to examine how impairment severity influences assistive benefit, while an individual with spinal muscular atrophy (SMA) was included to provide an additional reference for myoelectric signal characteristics in neuromuscular disease. These participants did not directly inform the design iterations but were used to probe generalization and to delineate the boundaries of applicability of the co-created system.

We have clarified this distinction in the revised manuscript by explicitly framing the co-creation process as impairment-specific and exploratory, and by positioning subsequent experiments as validation steps rather than extensions of the design process.

We added this sentence to the introduction: "The co-creation process was intentionally centered on a single individual with near-complete ALS-related hand paralysis to address the design requirements of severe impairment, while participants with other neurological conditions were included at later stages for validation and comparative analysis rather than to inform design iterations."

We also added this sentence to the result(subsection: Validation of hand function assistance and usability with stroke patients): "These experiments were designed to assess assistive performance across impairment levels and did not inform the exoskeleton's mechanical or con-

trol design, which was finalized during the preceding co-creation phase."

We also added a paragraph to the discussion, as this is inline with reviewer's 1 comment 2: "Within a translational framework, the single-patient co-creation study should be interpreted as both exploratory and demonstrative. The iterative design process, bio-inspired actuation strategies, and intentional control interface were explored and refined exclusively in close collaboration with one individual with near-complete ALS-related hand paralysis, and therefore primarily demonstrate feasibility rather than population-level efficacy. At the same time, the resulting ability to reliably perform diverse grasp types and to regain a meaningful activity of daily living, namely independent feeding, serves as a demonstrative proof of principle for what multi-articulate soft hand exoskeletons can enable when tailored to severe impairment. Findings related to control performance, usability, and functional gains should not yet be generalized beyond this impairment level or design configuration, motivating the subsequent validation with stroke patients and future studies with larger cohorts."

We also added a sentence to Materials and Methods (subsection: Patients): "The participant with spinal muscular atrophy (SMA) did not perform exoskeleton-assisted tasks and was included exclusively to provide comparative EMG signal data for neuromuscular disease, enabling contextualization of signal quality and onset detection performance."

Response for (ii): We agree with the reviewer that hand paralysis arising from different neurological etiologies can present distinct clinical manifestations, including spasticity following stroke and flaccid weakness in neuromuscular disorders such as ALS and SMA. The co-created exoskeleton was primarily designed to address severe loss of voluntary hand function, with a focus on enabling preshaping, grasp stabilization, and object manipulation in the absence of reliable voluntary control.

The current system does not explicitly target spasticity management or tone reduction, and we do not claim that the presented design is optimized for spastic hand postures commonly observed after stroke. Instead, stroke patients were included to evaluate how impairment severity and residual voluntary control influence assistive benefit, rather than to represent a comprehensive design solution for spastic paresis. The observed limited or negative effects in some moderately impaired stroke patients further underscore that assistive strategies optimized for flaccid or near-complete paralysis may not directly translate to conditions characterized by spasticity and abnormal synergies.

We have clarified this distinction in the revised manuscript by explicitly framing the proposed system as impairment-specific and by acknowledging that spasticity-related impairments may require different mechanical compliance, control strategies, or therapeutic integration, which are beyond the scope of the present study.

We added to discussion: "The limited or negative performance changes observed in moderately impaired stroke patients likely reflect a combination of factors rather than a failure of the assistive approach. Notably, the present design does not explicitly address spasticity or abnormal muscle tone, which are common after stroke and may require distinct mechanical

compliance, control strategies, or therapeutic integration.

Response for (iii): The participant with spinal muscular atrophy (SMA) was intentionally included only in the EMG experiments and did not perform exoskeleton-assisted tasks. The purpose of including this participant was to provide comparative myoelectric signal data from a neuromuscular disorder distinct from ALS, enabling contextualization of signal quality, signal-to-noise ratio, and onset detection performance across conditions characterized by severe muscle weakness.
Because the mechanical design and control strategies of the exoskeleton were finalized during the preceding co-creation process with the ALS participant, and because the SMA participant exhibited a different clinical presentation and functional needs, we did not conduct exoskeleton-assisted functional evaluations with this individual. We therefore do not claim functional validation of the exoskeleton in SMA, and we have clarified in the revised manuscript that the SMA participant served solely as an EMG reference rather than as a functional test subject.

We added a sentence to Materials and Methods (subsection: Patients): "The participant with spinal muscular atrophy (SMA) did not perform exoskeleton-assisted tasks and was included exclusively to provide comparative EMG signal data for neuromuscular disease, enabling contextualization of signal quality and onset detection performance."

Comment 2: *Second, the author commented that to be effective in daily living an exoskeleton "should be soft and lightweight, intuitively controlled, and capable of supporting the grasp of objects with varying shapes and weights". Several of these characteristics have not been demonstrated true in the current design. For instance, the description of the weight should be reported in the main results and compare to other similar devices. The "intuitively controlled" should be demonstrated comparing EMG control versus button trigger control in terms of motor performances over different participants and sessions. Testing of the exoskeleton control paired with some cognitive tasks is also often used to demonstrate the intuitive control of a device. Finally, no tests with objects with varying weights were performed.*

Response (general): We thank the reviewer for this detailed comment. To address it clearly, we respond separately to the points regarding (i) reporting and benchmarking of device weight, (ii) the interpretation of "intuitive control," (iii) the absence of cognitive dual-task testing, and (iv) evaluation across objects with different weights.

Response for (i): We thank the reviewer for highlighting the importance of device weight for daily use. The weight of the wearable hand exoskeleton (352 g) as well as the fully integrated control box (approximately 2 kg, including compressor, valve manifold, and electronics) is explicitly reported in the Materials and Methods section, Soft Robotic Hand Exoskeleton subsection. We have ensured that these values are clearly stated and easy to locate in the revised manuscript.
Because the presented system incorporates multiple pneumatic actuators, valves, sensors, and an onboard air supply to enable multi-articulate assistance and thumb opposition for individuals with severe hand paralysis, its overall weight is not directly comparable to simpler flexion–extension gloves, passive orthoses, or button-triggered devices that target different im-

pairment levels and functional goals. We therefore avoid claiming superiority in terms of absolute mass and instead position the system relative to existing approaches in terms of functional capability, dexterity, and target population, which we clarify in the Discussion.

Response for (ii): We thank the reviewer for this important clarification. In the revised manuscript, we have refined our use of the term "intuitive control" to avoid implying a systematic quantitative comparison across control modalities. In this work, "intuitive" refers specifically to the user's ability to initiate grasping through residual muscle activity in a hands-free manner, without relying on explicit external triggers such as buttons or switches, which are commonly used in assistive devices.

We do not want to claim that the presented EMG-based interface is superior to button-based or other control strategies in terms of motor performance, nor do we provide a multi-participant or multi-session comparison. Instead, we frame the EMG interface as an impairment-adapted intentional control strategy that was preferred by the co-creation participant due to its integration with natural motor intent and its ability to support spontaneous grasp initiation. We have revised the manuscript accordingly to ensure that this interpretation is clearly stated and appropriately scoped.

We replaced "intuitively controlled" in the Result section (subsection: Co-creation reveals patient priorities and engineering requirements for assisting individuals with severe hand impairments)
BY:
"hands-free and intention-driven (e.g., via residual myoelectric activity"

We also replaced "intuitively control the exoskeleton with low delay"in the Results section (subsection: Grasping intention can be detected from non-invasive EMG signals despite low signal-to-noise ratio)
BY: "control the exoskeleton in a hands-free manner with low delay, more natural than pressing a button,"

Response for (iii): We agree with the reviewer that cognitive dual-task paradigms are a useful and widely used approach to quantify intuitiveness and cognitive load of assistive device control. Such an evaluation was beyond the scope of the present study, which focused on demonstrating the feasibility of impairment-adapted intentional control, functional assistance, and usability in individuals with severe hand impairment.
Accordingly, we do not claim a quantified reduction in cognitive load or superior intuitiveness relative to alternative control modalities. To avoid overinterpretation, we have revised the manuscript to use more precise wording (e.g., hands-free, intention-driven control) and we explicitly identify cognitive dual-task testing as an important direction for future work in subsequent controlled clinical evaluations.

We updated the new paragraph, which was already added to answer Reviewer1 comment7,
BY:

"Future evaluations should also quantify user cognitive load and control intuitiveness using established dual-task paradigms."

Response for (iv): We agree with the reviewer that systematic evaluation across a wide range of object weights is important for assessing general-purpose assistance in activities of daily living. The present study was not designed as a comprehensive weight-benchmarking evaluation. Instead, our objective was to demonstrate the feasibility of restoring intentional grasping and object manipulation in individuals with severe to near-complete hand paralysis, for whom baseline functional capability is effectively zero.
Accordingly, we evaluated grasping using representative everyday objects, including bottles, dishes, cups, fruits, and standardized clinical assessment items (e.g., ARAT objects), whose weights are sufficient to demonstrate functional relevance and support against gravity, rather than performing a parametric sweep across object masses. We have revised the manuscript to clarify this scope and to avoid implying general activities-of-daily-living coverage across arbitrary object weights. Systematic evaluation across object weight distributions remains an important direction for future controlled studies and clinical translation.

In results (subsection: Co-creation enables customization for severe impairment), **we replaced** "demonstrating the capability of the hand exoskeleton to support multiple grasp types for diverse objects of everyday life"
BY:
"demonstrating the capability of the hand exoskeleton to support multiple grasp types for representative everyday objects relevant to daily living tasks."
Motivation: Remove implication of exhaustive ADL coverage.

In Fig.1 caption C, **we replaced** "intentional grasping of objects of everyday life with diverse shapes and weights"
BY:
"intentional grasping of representative everyday objects with diverse shapes and functionally relevant weights"
Motivation: Demonstrate functional relevance rather than completeness.

In Discussion, **we added** "Future studies should systematically evaluate performance across broader object weight ranges to quantify load limits and generalization to diverse daily tasks."
Motivation: Position weight benchmarking as future work.

Comment 3: *Finally, while the author did a very good job on showing how the exoskeleton can improve performances in the BBT task, the manuscript lacks data on supporting reliability and robustness of the results. Here a few examples where more data would be necessary to support the authors conclusions. For instance, in the tests with the 2 intact participants, the authors reported that all grasp types resulted in more than 125 successful grasp-and-lift actions. However, it is unclear how many times did the grasps fail. Moreover, it would be interesting to see the reliability of repetitions of the pre-shaping and grasping. Can the exoskeleton detect and replicate these positions over several repetitions? It would also be important to see how positioning of the hand*

(for instance movement against vs. towards gravity) affects performances. The same analysis (maybe with a few less grasp/hand gesture because of fatigue) should be repeated for the patients. Indeed, patients' performances are often very variable, and this can affect robot performances. Moreover, it would be important to understand whether the EMG control would need to be recalibrate over sessions. This is usually a big drawback of EMG/neural control systems. The authors also reported that the "the co-created design changes enabled the ALS participant to reliably grasp a bottle (cylindrical grasp), a plate (lateral grasp), an apple (spherical grasp), and a pastry (pinch grasp), thus demonstrating the capability of the hand exoskeleton to support multiple grasp types for diverse objects of everyday life. None of these data were reported in the manuscript. Finally, it is unclear why stroke participants have not been tested in similar daily living tasks.

Response (general): We thank the reviewer for this comprehensive comment regarding robustness, reliability, and reproducibility. To ensure clarity, we address the different aspects separately below, including (i) reporting of grasp success and failure rates, (ii) repeatability of preshaping and grasp execution, (iii) effects of hand orientation and gravity, (iv) robustness and recalibration of EMG control across sessions, (v) reporting of everyday object grasping results, and (vi) the absence of daily living task evaluations in stroke participants.

Response for (i): Below we present a table showing for each grasp type the total number of grasp attempts, successful grasps and failed grasps during our grasp robustness experiment.

Table 1: Grasp robustness experiment results

Grasp type	Total attempts	Successful grasps	Failed grasps	Overall acc
Pinch	167	159	8	95.2%
Cylindrical	128	125	6	97.7%
Spherical	132	126	6	95.5%
Lateral	138	132	6	95.7%

We have added this table to the results subsection "Thumb dexterity enables reliable grasping".

Response for (ii):

To address repeatability of preshaping and grasp execution, we have added a new supplementary video (see Fig.1) demonstrating repeated execution of preshaping and grasping across multiple ARAT-relevant objects using the silicone hand model. This model was intentionally employed to eliminate confounding contributions from human motor variability and to isolate the repeatability of the exoskeleton's actuation and control.

The video shows that the exoskeleton consistently reproduces the same preshaping configurations and grasp strategies across repetitions, including hand aperture, thumb opposition, and grasp closure, without manual retuning. These demonstrations support functional repeatability of the pneumatic actuation and control strategy under controlled conditions. We clarify in the revised manuscript that this assessment focuses on mechanical and control repeatability, while quantitative kinematic analysis and patient-level repeatability remain important directions for future work.

We added this sentence to the results section (subsection: Validation of hand function

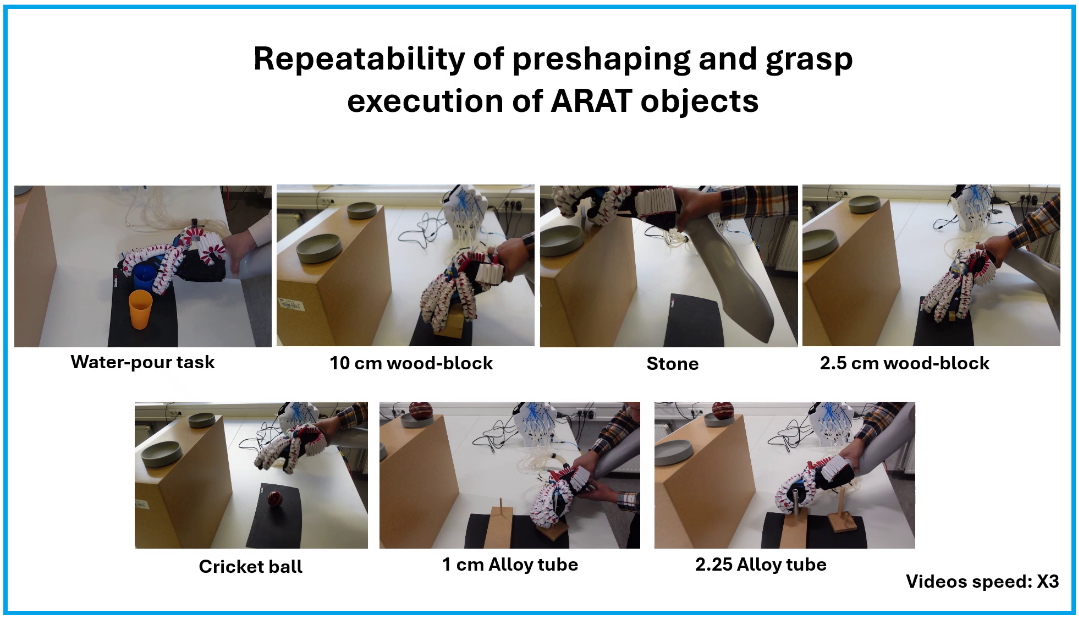

Figure 1: Extracted from the repeatability video.

assistance and usability with stroke patients): "Repeatability of preshaping and grasp execution across multiple repetitions and ARAT-relevant objects, evaluated using a passive silicone hand model to isolate device behavior, is illustrated in Supplementary Video"Repeatability-ARAT"."

Response for (iii):

We agree with the reviewer that hand orientation and movement relative to gravity can substantially affect grasp performance. While we did not perform a systematic orientation-controlled study, the robustness experiments and the newly added supplementary video inherently include variations in hand orientation and gravitational loading.

In particular, the supplementary video includes a water-pouring task, which requires stable grasp formation, lifting against gravity, controlled wrist orientation, and maintenance of grasp stability during dynamic load changes while pouring. This task therefore provides a representative example of grasp robustness under changing gravitational and inertial conditions. Across repeated executions, the exoskeleton maintained stable preshaping and grasp control throughout lifting, pouring, and placement phases.

Additional demonstrated tasks involve lifting and placing objects of different geometries with varying hand orientations, further supporting robustness to moderate orientation-dependent loading. We clarify in the revised manuscript that these demonstrations provide qualitative evidence of robustness, while systematic evaluation of orientation-specific performance remains an important direction for future work.

We extended the previously added sentence by "including a water-pouring task that demonstrates stable grasping during lifting, wrist reorientation, and dynamic load changes.", **so it becomes as:** "Repeatability of preshaping and grasp execution across multiple repetitions and ARAT-relevant objects, evaluated using a passive silicone hand model to isolate device behavior, is illustrated in Supplementary Video "Repeatability-ARAT", including a water-pouring task that demonstrates stable grasping during lifting, wrist reorientation, and dynamic load

changes."

Response for (iv): We thank the reviewer for raising this important point. We acknowledge that recalibration across sessions is a well-known challenge for EMG/neural control systems. However, our results in the section "Grasping intention can be detected from non-invasive EMG signals despite low signal-to-noise ratio" provide evidence for a degree of cross-session and cross-subject robustness in our trained classifiers. Specifically, when we trained the 1D CNN onset detection model on EMG-RMS data from the 15 healthy participants, which were recorded on different days and with small shifts in electrode placement, albeit over the same target muscle, and evaluated it on the ALS patient's EMG data, we still achieved 100% sensitivity and 90.6% specificity. Similarly, adding data from the individual with SMA maintained the high sensitivity while slightly increasing specificity to 90.9%. These results suggest that the learned representations generalize reasonably well across recording sessions, subjects, and conditions, despite the substantial differences in motor ability. That said, the best performance was obtained when training on the ALS patient's own data (100% sensitivity, 96.7% specificity), which suggests a personalization effect and indicates that session-specific recalibration can further improve performance. For our co-creation sessions, we addressed day-to-day variability pragmatically through the adaptive thresholding approach, which could be quickly adjusted to the patient's daily condition without requiring data collection and model retraining. We note that the field has made significant progress in recent years in mitigating the recalibration burden, particularly through large-scale data-driven generalization across users (Kaifosh et al., 2025, Nature) and unsupervised online adaptation and context-informed incremental learning (Campbell et al., 2024, Journal of NeuroEngineering and Rehabilitation), which could be integrated into the proposed system in future iterations to further enhance the robustness of our EMG-based exoskeleton control.

We added these considerations and relevant research directions to the results section "Grasping intention can be detected from non-invasive EMG signals despite low signal-to-noise ratio": "Notably, the retention of high sensitivity when training on healthy participant data and evaluating on the ALS patient's EMG, despite differences in recording sessions, electrode placement, and neuromuscular pathology, suggests a degree of cross-session and cross-subject robustness in the learned onset representations. This is consistent with recent findings that EMG decoding models can generalize across users and sessions when trained on sufficiently large datasets (Kaifosh et al., 2025). Furthermore, advances in unsupervised online adaptation (Campbell et al., 2024) offer promising strategies to further reduce recalibration burden in future system iterations, particularly for progressive conditions such as ALS where signal characteristics change over time."

Response for (v) and (vi): Throughout the revision, we have reduced emphasis on activities of daily living as a primary outcome and clarified that ADL examples serve as illustrative context rather than validated performance endpoints.

We replaced the abstract text "The exoskeleton assistance enabled the patient to intentionally grasp everyday objects, achieve a Box-and-Blocks Test score of 5, and regain the ability

to feed himself" **BY:** "The exoskeleton assistance enabled the patient to intentionally grasp objects, achieve a Box-and-Blocks Test score of 5, and perform functionally meaningful object manipulation, including feeding himself."

We added to Results section (subsection: Validation of hand function assistance and usability with stroke patients) "Validation focused on standardized clinical assessments of hand function rather than task-specific daily living activities".

We also added to discussion: "While activities of daily living provide an important motivational context, the present study evaluates assistive benefit primarily through standardized clinical measures and functional grasping tasks, rather than comprehensive ADL performance."

Comment 4: *Minor comment: The organization of the Introduction is somewhat unusual. It begins by presenting the problem, then introduces the authors' solution, and subsequently returns to the problem statement. This structure makes the narrative difficult to follow. In addition, including figures in the Introduction is uncommon. I suggest revising this section to improve linearity and clarity.*

Response: We thank the reviewer for this constructive comment regarding the organization of the Introduction. We have revised the Introduction to improve linearity and remove the previously observed "problem–solution–problem" zig-zag structure. Specifically, we repositioned the paragraph describing the requirements of severely impaired patients, namely the need for increased dexterity, hand preshaping, and an active opposable thumb, so that it now directly follows the general overview of existing soft hand exoskeletons and their limitations. We believe this restructuring improves narrative clarity and better motivates the design choices presented in the manuscript.

Response to the Reviewers

Manuscript Title:
A Dexterous Soft Hand Exoskeleton Restores Intentional Grasping for Individuals with Severe Hand Impairment

Journal: Nature Machine Intelligence

We thank the Editor and the Reviewers for their careful evaluation of our manuscript and for their constructive and insightful comments. We are pleased that both reviewers found the work to be of interest and recognized the translational relevance, co-creation methodology, and clinical potential of our approach. Below, we address each comment point by point. All changes are reflected in the revised manuscript with tracked changes enabled, and relevant section references are provided for clarity.

Response to Reviewer #1

The paper reports the co-design and evaluation of a multi-articulate, pneumatically actuated soft hand exoskeleton targeting individuals with severe to near-complete hand paralysis, primarily an ALS patient with a pre-intervention BBT score of zero. The central technical contribution is the integration of an active opposable and abductable thumb with wrist dorsiflexion support, filling a well-documented gap in the soft exoskeleton literature, where most devices are limited to simple flexion-extension and target moderately impaired users. The paper has been significantly improved. Here some additional issues:

Comment 1: *As pointed out, the entire design process rested on a single ALS patient, and that stroke-related spasticity presents very different mechanical and control requirements. The authors' response is honest: they reframe the co-creation study as feasibility-demonstrative rather than population-representative, explicitly carve out stroke patients and the SMA participant as validation rather than co-design contributors, and add appropriate caveats in the abstract, introduction, and discussion. This is the right framing, though it also makes the claim of "broader applicability" correspondingly more modest. The authors should work on this.*
Response: We thank the reviewer for this important comment. In line with this suggestion, we have revised the manuscript to remove implications of broad generalizability and to better align our claim with the scope of the study.

In the abstract, we removed "To evaluate broader applicability"

Comment 2: *Robustness and reliability of the data was an issue we raised before. The authors add a grasp success/failure table (overall accuracy 95–98% across grasp types in healthy controls), a repeatability supplementary video including a water-pouring task, and cross-session EMG generalization analysis. These additions meaningfully strengthen the paper. However, no patient-level repeatability data is provided across sessions, which remains an important open question, particularly given the progressive nature of ALS and the acknowledged need for periodic threshold recalibration.*

Response: We thank the reviewer for recognising the improvements made to address robustness and reliability. We agree that patient-level repeatability across sessions represents a valuable next step, and the progressive nature of ALS makes this a particularly meaningful question. In the course of our study, we had to progressively lower the detection threshold as the ALS patient's muscles weakened. While this allowed us to keep the sensitivity high, it increased false positives. As noted in the paper, error monitoring and correction enabled us to reduce the error rate and improve reliability, enabling the patient to successfully perform the box-and-blocks test across sessions.

We extended our discussion to further emphasize this open research question: "[...] the intentional control interface requires individual calibration and periodic adjustment to accommodate day-to-day variability in residual muscle activity. Particularly in progressive conditions such as ALS, longitudinal repeatability is of high clinical relevance. While threshold adjustments in combination with error monitoring and correction can help to counteract disease-related EMG signal reduction, developing and evaluating methods for improving cross-session reliability, particularly over long time horizons, remains an important open research question.

Comment 3: *Claims about intuitiveness and weight: the authors appropriately remove the term "intuitively controlled" and replace it with "hands-free and intention-driven," and acknowledge the absence of cognitive dual-task validation. This is a reasonable scope correction. The weight issue (352 g glove + 2 kg control box) is acknowledged as a deployment barrier, though the 2 kg compressor-valve-electronics unit is a non-trivial portability limitation that may deserve more emphasis given the intended home-use aspiration.*
Response: We thank the reviewer for this insightful comment. We agree that the portability of the control unit represents a non-trivial limitation, particularly in the context of intended home use. In response, we have revised the discussion to explicitly emphasize this point.

In the Discussion, we replaced "Second, pneumatic actuation, while lightweight, compliant, and well suited for soft wearable devices, requires an integrated air supply and introduces constraints related to portability, noise, and long-term robustness that must be addressed for routine clinical or home use."

BY

"Second, pneumatic actuation, while lightweight, compliant, and well suited for soft wearable devices, requires an integrated air supply. In the current system, this results in a compressor–valve–electronics unit weighing approximately 2 kg, which constitutes a non-trivial limitation in terms of portability. While the glove itself is lightweight, the overall system currently restricts fully independent use and represents a significant barrier to deployment in unsupervised home environments. Reducing the size, weight, and power requirements of this control unit is therefore a key requirement for future translation."

At the end of the Discussion, we replaced "improving portability"

BY

"substantially reducing system size and weight to enable independent home use"

Comment 4: *The most significant unresolved limitation is that the exoskeleton is, by construction, calibrated to one individual's anatomy, residual function, and disease stage. The nine-month co-creation process is a strength methodologically but also means that design choices — thumb geometry, actuator pressures, electrode placement — may not transfer without significant re-engineering to other ALS patients, even at comparable disease stages. The validation cohort (n=6 stroke patients, one ALS co-creator) is too small to draw population-level conclusions, which the authors now acknowledge explicitly.*
Response: We thank the reviewer for highlighting this important limitation. We agree that the presented system is highly individualized and that its performance relies on calibration to the specific anatomy, residual motor function, and disease stage of the co-creation participant. In response, we have further strengthened the Discussion to explicitly state that key design parameters, including thumb geometry, actuator pressures, and electrode placement, were tailored to a single individual and are not expected to directly transfer to other patients without adaptation. We clarify that even among individuals with comparable ALS severity, differences in musculoskeletal structure and residual control signals may require substantial re-engineering of both the mechanical design and the control interface.
We also emphasize that the present study should be interpreted as a feasibility-demonstrative and exploratory investigation, rather than evidence of population-level efficacy. While the validation with stroke patients provides insight into assistive performance across impairment levels, the cohort size remains insufficient to support generalizable conclusions.
These clarifications ensure that the scope and limitations of personalization and transferability are explicitly and consistently communicated.

We added to the discussion, "The presented system is highly individualized, and key design parameters, including actuator configuration, thumb geometry, open or closed palm, intentional control approach, and EMG electrode placement, were tailored to a single participant and are not expected to transfer directly to other patients without substantial adaptation. Nevertheless, as they emerged as design priorities from a structured co-design process grounded in clinical and user evaluation, they offer a documented rationale that may guide personalization for other patients."

Response to Reviewer #2

I would like to thank the authors for adressing all my comments and modifying the manuscript accordingly. There is still one point that needs to be addressed. I appreciate the addition of the supplementary video to show repeatability of preshaping and grasp execution.

Comment 1: *However, I believe it would be important to quantify how replicable and successful were the different tasks.*

Response: We thank the reviewer for this suggestion. We have added quantitative results to the Results section to explicitly report the repeatability and success of the tasks shown in the supplementary video.

We added to the Result section: "Each task was repeated for 10 trials, with successful grasp execution achieved in all repetitions. Task completion times were consistent across repetitions, with average execution times of 7.87 ± 1.12 s (water pouring), 5.42 ± 0.40 s (10 cm block), 5.05 ± 0.77 s (stone), 1.99 ± 0.23 s (2.5 cm block), 2.87 ± 0.29 s (cricket ball), 5.52 ± 0.89 s (1 cm tube), and 4.63 ± 0.47 s (2.25 cm tube). These reported execution times were computed from the predefined motor command sequences. The start of each trial was defined as the instant corresponding to the first actuator activation ($t = 0$), triggered by the operator of the dummy arm, while the end time was determined by the completion of the final release phase, also initiated by the operator via the control interface. This introduces a limited user-dependent component in the timing, associated with the initiation and termination of the sequence. However, once triggered, the execution follows a deterministic motor program with millisecond resolution, ensuring consistency across repetitions. The measured durations therefore reflect the combined effect of the programmed coordination of actuator commands, the intrinsic pneumatic response of the system, including valve dynamics and pressure evolution, and the operator's interaction during task execution."
